# Comparing Effects of Transforming Growth Factor β1 on Microglia From Rat and Mouse: Transcriptional Profiles and Potassium Channels

**DOI:** 10.3389/fncel.2018.00115

**Published:** 2018-05-03

**Authors:** Starlee Lively, Doris Lam, Raymond Wong, Lyanne C. Schlichter

**Affiliations:** ^1^Krembil Research Institute, Genes and Development Division, University Health Network, Toronto, ON, Canada; ^2^Department of Physiology, University of Toronto, Toronto, ON, Canada

**Keywords:** microglia, rat, mouse, TGFβ1, potassium channels, transcription profiling, microglial activation, anti-inflammatory

## Abstract

The cytokine, transforming growth factor β1 (TGFβ1), is up-regulated after central nervous system (CNS) injuries or diseases involving microglial activation, and it has been proposed as a therapeutic agent for treating neuroinflammation. Microglia can produce and respond to TGFβ1. While rats and mice are commonly used for studying neuroinflammation, very few reports directly compare them. Such studies are important for improving pre-clinical studies and furthering translational progress in developing therapeutic interventions. After intracerebral hemorrhage (ICH) in the rat striatum, the TGFβ1 receptor was highly expressed on microglia/macrophages within the hematoma. We recently found species similarities and differences in response to either a pro-inflammatory (interferon-γ, IFN-γ, +tumor necrosis factor, TNF-α) or anti-inflammatory interleukin-4 (IL-4) stimulus. Here, we assessed whether rat and mouse microglia differ in their responses to TGFβ1. Microglia were isolated from Sprague-Dawley rats and C57BL/6 mice and treated with TGFβ1. We quantified changes in expression of >50 genes, in their morphology, proliferation, apoptosis and in three potassium channels that are considered therapeutic targets. Many inflammatory mediators, immune receptors and modulators showed species similarities, but notable differences included that, for some genes, only one species responded (e.g., *Il4r*, *Il10*, *Tgfbr2*, colony-stimulating factor receptor (*Csf1r*), *Itgam*, suppressor of cytokine signaling 1 (*Socs1*), toll-like receptors 4 (*Tlr4*), *P2rx7*, *P2ry12*), and opposite responses were seen for others (*Tgfb1*, *Myc*, *Ifngr1*). In rat only, TGFβ1 affected microglial morphology and proliferation, but there was no apoptosis in either species. In both species, TGFβ1 dramatically increased Kv1.3 channel expression and current (no effects on Kir2.1). KCa3.1 showed opposite species responses: the current was low in unstimulated rat microglia and greatly increased by TGFβ1 but higher in control mouse cells and decreased by TGFβ1. Finally, we compared TGFβ1 and IL10 (often considered similar anti-inflammatory stimuli) and found many different responses in both species. Overall, the numerous species differences should be considered when characterizing neuroinflammation and microglial activation *in vitro* and *in vivo*, and when targeting potassium channels.

## Introduction

In the central nervous system (CNS), transforming growth factor β1 (TGFβ1) is important for development of microglia (Butovsky et al., 2014), and is thought to help maintain their “resting” state (Spittau et al., 2013). TGFβ1 is up-regulated after CNS damage or in disease states that involve microglial activation, including stroke and neurodegenerative diseases (Lehrmann et al., 1998; Lively and Schlichter, 2012; Lively et al., 2016; Chen et al., 2017; Taylor et al., 2017). TGFβ1 is expressed in microglia in the healthy brain (De Groot et al., 1999) as is the TGFβ1 receptor (De Groot et al., 1999; Pál et al., 2014). After experimental stroke, TGFβ1 (Wiessner et al., 1993; Lehrmann et al., 1998) and the TGFβ1 receptor (Pál et al., 2014) are up-regulated, especially in microglia/activated macrophages. Brain TGFβ1 also rapidly increased in a mouse model of intracerebral hemorrhage (ICH; Taylor et al., 2017). To improve the use of animal models for translation to human CNS diseases, it is important to determine whether immune responses are conserved among species. As a prelude to this *in vitro* study, we first determined that TGFβ1 mRNA (Lively and Schlichter, 2012) and the TGFβ1 receptor protein (present study) increased in a rat model of ICH.

There are surprisingly few studies comparing microglial responses in these rodent species. Our recent study showed many differences in responses of rat and mouse microglia to pro- and anti-inflammatory cytokines (Lam et al., 2017). Based on microglial responses to TGFβ1 *in vitro* and *in vivo*, it is thought that this cytokine promotes a homeostatic phenotype (Abutbul et al., 2012; Spittau et al., 2013; Butovsky et al., 2014; Krasemann et al., 2017). The microglial reaction after CNS damage is often followed by resolution and repair phases, and many authors have defined a microglial “acquired deactivation” state, similar to macrophage responses to TGFβ1, interleukin-10 (IL-10) or glucocorticoids (Mantovani et al., 2004; Boche et al., 2013; Cherry et al., 2014; Orihuela et al., 2016). Recently, some transcriptional profiling of TGFβ1 responses has been published for mouse microglia (Butovsky et al., 2014; Taylor et al., 2017), and two studies compared a small number of molecular responses to TGFβ1 and IL-10 in mouse (Ledeboer et al., 2000) and rat microglia (Lodge and Sriram, 1996). However, more detailed comparisons of both species are needed. Here, we compared transcriptional profiles of rat and mouse microglia in response to TGFβ1 application *in vitro* by quantifying >50 genes that included microglial activation markers, pro- and anti-inflammatory mediators and receptors, signaling molecules, nicotinamide adenine dinucleotide phosphate (NADPH) oxidases (NOX enzymes), purinergic and phagocytic receptors, as well as several ion channels and regulators. At the protein level, we also assessed the TGFβ1 receptor (TGFβR1), the interferon-γ (IFNγ) receptor (IFNGR1), the mannose receptor (CD206/MRC1) and the Ca^2+^-activated K^+^ channel, KCa3.1. While there were some species similarities in responses to TGFβ1, the observed differences reinforce the importance of analyzing both species in pre-clinical studies. Moreover, in comparing the TGFβ1 responses with our recently published analysis of IL-10 responses of primary rat and mouse microglia, substantial differences were seen between these two anti-inflammatory cytokines.

When considering microglial contributions to CNS damage and recovery, numerous recent studies using specific K^+^ channel blockers have shown roles for Kv1.3, Kir2.1 and KCa3.1 channels, leading to their consideration as targets for regulating neuroinflammation (for recent reviews and further references, see Lam and Schlichter, 2015; Lam et al., 2017; Nguyen et al., 2017a,b). In principle, K^+^ channels hyperpolarize cells and increase the driving force for Ca^2+^ entry. Previous work from our lab (for instance, Jiang et al., 2003; Ohana et al., 2009; Ferreira et al., 2014; Siddiqui et al., 2014; Lam and Schlichter, 2015) and others (e.g., Heo et al., 2015; Michaelis et al., 2015) shows that microglia use Ca^2+^ entry through store-operated channels (SOC) and Ca^2+^-release-activated Ca^2+^ (CRAC) channels to regulate many microglial functions, including proliferation, migration, phagocytosis and cytokine production (reviewed in Möller, 2002; Färber and Kettenmann, 2006). Therefore, we compared transcript expression between rat and mouse microglia, with and without TGFβ1 treatment, for several Ca^2+^ and K^+^ channels and regulators; as well as current amplitudes (the final outcome of functioning channel proteins) for Kir2.1, Kv1.3, and KCa3.1. Again, despite some similar responses, there were species differences that should be considered for future translational studies.

## Materials and Methods

All procedures on animals adhered to the Canadian Council on Animal Care guidelines for humane animal use and were approved by the University Health Network Animal Care Committee (Animal Use Protocols 914, 1573 and 1132).

### Staining Intracerebral Hemorrhage Tissue

The methods for creating an ICH by injecting collagenase into the striatum of male Sprague-Dawley rats (300–350 g; Charles River, Saint-Constant, QC, Canada), and preparing the tissue and cryosections for immunohistochemistry were exactly as in our recent work (Moxon-Emre and Schlichter, 2011; Lively and Schlichter, 2012; Joseph et al., 2016). Microglia/macrophages were labeled with antibodies against CD11b (OX42, 1:200, Bio-Rad/AbD Serotec, RRID:SCR_013553; Cat# MCA275G, RRID:AB_321301) and TGFβR1 (1:500, Abcam, RRID:SCR_012931; Cat# ab31013, RRID:AB_778352). Secondary antibodies were Alexa-conjugated and used at 1:200 dilution (Jackson ImmunoResearch Labs, West Grove, PA; Cat# 715-545-150, RRID:AB_2340846; Cat# 711-585-152, RRID:AB_2340621) in phosphate buffered saline (PBS; Wisent, Saint-Jean-Baptiste, QC, Canada; Cat# 311-010-CL) with 0.2% Triton X-100 (Sigma-Aldrich, RRID:SCR_008988; Cat# T8787) and 5% donkey serum (Jackson ImmunoResearch Labs; Cat# 017-000-121). To label cell nuclei, 4’-6-diamidino-2-phenylindole (DAPI; 1:5000; Sigma-Aldrich; Cat# D9542) was applied for 10 min. Slides were mounted in Dako mounting medium (Agilent-Dako, RRID:SCR_013530; Cat# S302380-2) and stored in the dark at 4°C. Images were obtained with a confocal microscope (model LSM880; Zeiss, Oberkochen, Germany) and processed using ImageJ software, version 1.47v^1^(RRID:SCR_003070).

### Isolating Microglia and Treating Them With TGFβ1

#### Neonatal Microglia

Microglia were isolated from Sprague-Dawley rat pups (P1–P2) and C57BL/6 mouse pups (P0–P2; both from Charles River), as recently described (Lam et al., 2017). These strains were selected because they are widely used, and C57BL/6 is the main strain for transgenic studies. In brief, mashed brain tissue was strained and centrifuged (300× *g*, 10 min) in cold Minimal Essential Medium (MEM; Thermo Fisher Scientific, RRID:SCR_008452; Cat# 11095080). Mixed brain cell cultures were established by re-suspending the pellet in MEM, incubating in 75 cm^2^ flasks with 20 ml MEM supplemented with 10% fetal bovine serum (FBS; Wisent; Cat# 080-150) and 0.05 mg/ml gentamycin (Thermo Fisher Scientific; Cat# 15710072). The medium was changed after 2 days, and microglia (≥99% pure) were harvested a few days later by shaking the flasks (5 h, 65 rpm) on an orbital shaker in the incubator, centrifuging the supernatant, and re-suspending the cells in fresh MEM (with 2% FBS, 0.05 mg/ml gentamycin). Microglia were seeded onto UV-irradiated 15 mm coverslips (Fisher Scientific, Ottawa, ON, Canada; Cat# 12-545-83) at densities that are noted under each assay. Their initial state was similar for both species, as confirmed from the expression of numerous genes (see “Results” section).

#### Adult Microglia

Adult male Sprague-Dawley rats (300–350 g) or C57BL/6J mice (20–30 g; Charles River) were deeply anesthetized with isoflurane and decapitated. After 400 mg of cerebral cortex was harvested, it was chopped into small pieces in 1 ml HBSS without Ca^2+^ or Mg^2+^ (Thermo Fisher; Cat# 14170112), and centrifuged (300× *g*, 2 min) to pellet the tissue. Following the manufacturer’s protocol for the Neural Dissociation Kit (P; Miltenyi Biotec, RRID:SCR_008984; Cat# 130-092-628), the supernatant was removed, cells were dissociated using Pasteur pipettes. To trap myelin, cells were incubated with myelin removal beads (Miltenyi Biotec; Cat# 130-096-733) at 4°C for 15 min, washed in PBS containing 0.5% bovine serum albumin (BSA; Bioshop, Burlington, ON, Canada; Cat# ALB001), centrifuged, resuspended in PBS/BSA buffer, and passed through LS columns (Miltenyi Biotech; Cat# 130-042-401). Eluted cells were centrifuged, resuspended in PBS/BSA buffer, and incubated at 4°C for 15 min with either human/mouse CD11b or rat CD11b/c microbeads (Miltenyi Biotech; Cat# 130-093-634 and 130-105-634, respectively). To collect eluted microglia, the cell suspension was washed, centrifuged, and resuspended in PBS/BSA buffer, and passed through MS columns (Miltenyi Biotech; Cat# 130-042-201). Microglia were centrifuged, resuspended in fresh MEM (with 2% FBS, 0.05 mg/ml gentamycin), seeded at 8 × 10^4^ cells/coverslip, and grown for 1–2 days before treating with TGFβ1.

Neonatal and adult microglia were left untreated (control; CTL) or treated with TGFβ1, which was diluted from a stock solution in sterile PBS with 0.3% BSA that was stored at −20°C. The mature form of the cytokine binds to its receptor, and the amino acid sequences for mature rat, mouse and human TGFβ1 are 99%–100% identical. Therefore, either mouse or human recombinant TGFβ1 could be used. Mouse cells were treated with recombinant mouse TGFβ1 (rmTGFβ1; R&D Systems Inc., RRID:SCR_006140; Cat# 7666-MB), and rat cells were treated with recombinant human TGFβ1 (rhTGFβ1; R&D Systems Inc.; Cat# 240-B) because a rat cytokine was not commercially available. rhTGFβ1 has often been used on rodent cells (e.g., Merrill and Zimmerman, 1991; Constam et al., 1992; Butovsky et al., 2014). Widely varying TGFβ1 concentrations have previously been used on microglia (0.001–100 ng/ml), but the type (species, purified, recombinant) was not always specified. For instance, 1–100 ng/ml of an unspecified type of TGFβ1 was needed to see effects on gene expression and cytokine production (Suzumura et al., 1993). hTGFβ1 was used on mouse cells at 10 ng/ml (Xiao et al., 1997; Schilling et al., 2000; Schilling and Eder, 2003) or 50 ng/ml (Butovsky et al., 2014), and on both species at 0.1 ng/ml (Merrill and Zimmerman, 1991; Constam et al., 1992). In the past, outcomes have usually been examined from 24 h to 72 h after treatment (e.g., Suzumura et al., 1993; Xiao et al., 1997; Butovsky et al., 2014). Therefore, we first conducted a pilot study on rat microglia with 6 or 24 h treatment at 1, 5, 10 or 20 ng/ml rhTGFβ1, and then selected 24 h for most experiments. Some 6 h results are mentioned in the text.

### Microglia Morphology, Proliferation and Apoptosis

#### Morphology

Microglial morphology and proliferation were examined, as before (Ferreira et al., 2014; Lam and Schlichter, 2015; Lam et al., 2017). For imaging, cells were seeded at 8 × 10^4^ cells/coverslip, stimulated with TGFβ1 for 24 h, and fixed in 4% paraformaldehyde (PFA; Electron Microscopy Sciences, Hatfield, PA, USA; Cat# 15710) for 10 min, permeabilized with 0.2% Triton X-100 for 5 min, washed, and labeled overnight with CD11b (1:2500, Bio-Rad/AbD Serotec). The next day, cells were washed in PBS and incubated for 1 h with Alexa 594-conjugated secondary antibody (Jackson ImmunoResearch Labs; Cat# 715-585-151, RRID:AB_2340855; 1:200). Cells were counterstained for 1 h with Acti-stain 488 phalloidin (1:50; Cytoskeleton Inc., RRID:SCR_013532; Cat# PHDG1-A) to visualize filamentous (F-) actin, and with DAPI (1:3000, 10 min) to label nuclei. Coverslips were mounted on glass slides using Dako mounting medium and stored in the dark at 4°C until images were acquired with a confocal microscope (model LSM880; Zeiss) and processed in ImageJ. Unipolar cells (those with a lamellum and trailing uropod) were counted using ImageJ in three micrographs each (20× magnification) and expressed as a percentage of total cells counted (~120–200/culture).

#### Proliferation

Proliferation was quantified using the CyQUANT™ NF assay (Thermo Fisher Scientific; Cat # C35006), as before (Lam and Schlichter, 2015). Microglia were seeded at 2–3 × 10^4^ cells/well of a 96-well flat-bottom plate in MEM with 2% FBS and allowed to settle for 24 h, then left untreated (control) or stimulated with TGFβ1 for 24 h. The detection dye was added to each well for 30 min (37°C, 5% CO_2_), and the fluorescence intensity was measured using a multi-label plate reader (Victor^3^ 1420, Perkin Elmer, Woodbridge, ON, Canada), with excitation at 485 nm and emission at 535 nm. Duplicate readings were taken for 0.1 s at 3 mm from the bottom of the plate, averaged, and then the background was subtracted before normalizing to the control group.

#### Apoptosis

Cells were seeded at 4 × 10^4^ cells/well in 96-well plates (as for proliferation, above), treated with TGFβ1 for 24 h, and fixed in 4% PFA for 7 min, washed in PBS and post-fixed in methanol for 20 min. Apoptotic cells were detected using the TiterTACS™ Colorimetric Apoptosis Detection Kit (Trevigen Cell Assays, Gaithersburg, MD, RRID:SCR_012449; Cat# 4822-96-K), with positive controls (TACS-Nuclease™ added for 10–20 min to generate DNA breaks) and negative controls (omitting TdT enzyme). Absorbance was measured at 450 nm using a SPECTROstar Nano plate reader (BMG LABTECH, Offenburg, Germany).

### NanoString Gene Expression Analysis

Microglia were seeded at 5 × 10^5^ cells/coverslip in a 12-well culture plate, allowed to settle for 1–2 days (37°C, 5% CO_2_), and then left untreated (control) or stimulated with TGFβ1 for 24 h. In a pilot study on primary rat microglia, some genes were also analyzed at 6 h after TGFβ1 treatment and the relevant results are mentioned in the text. The sample preparation and transcriptional profiling were conducted as before (Ferreira et al., 2014; Siddiqui et al., 2014, 2016; Lam et al., 2017). Microglia were stimulated with TGFβ1 for 24 h, and then total RNA was extracted using TRIzol reagent (Thermo Fisher Scientific; Cat# 15596018), purified using an RNeasy Mini Kit (QIAGEN, Mississauga, ON, Canada; Cat# 74104), and stored at −80°C. Extracted RNA (200 ng per sample) was sent to the Princess Margaret Genomics Centre (Toronto, ON, Canada^2^) where the assay was conducted (hybridization, detection, scanning). Separate plates had to be used for each species, with different probe sets that were designed and synthesized by NanoString nCounter technologies (see Supplementary Tables S1, S2 for probe sequences). Because gene names sometimes differ between species, for clarity, rat names are used.

Data were analyzed with nSolver Analysis Software (ver3.0; RRID:SCR_00342). Each transcript of interest was recognized by a capture probe and a reporter probe, and assay variability was minimized using negative reporter probes for background subtraction. Positive control reporter probes were included to assess the hybridization efficiency and detection range, and to calculate a scaling factor that was applied to all mRNA counts in each sample. Finally, a reference gene scaling factor was calculated in the same manner using the housekeeping genes, *Hprt1* and *Gusb*, and used to adjust the counts for each sample (control, TGFβ1 treated).

### Western Blot Analysis

Protein changes generally map well onto mRNA changes, which we previously validated for several molecules investigated here (iNOS, COX-2, Pyk-2, CD206, Arg1, Iba1; Lam et al., 2017), and here, we examined four proteins using the same methods. Microglia were seeded on 25 mm coverslips (Fisher Scientific, Ottawa, ON, Canada; Cat# 12-545-86) in 35 mm culture dishes at 1–3 × 10^6^ cells (4–7 independent cell cultures for rat; 4–8 for mouse). Cells were incubated for 24 h with or without TGFβ1, and then briefly washed with PBS, lysed for 30 min in ice-cold RIPA lysis buffer with a mammalian protease inhibitor cocktail (Sigma-Aldrich; Cat# P3840), and spun down to remove insoluble material. Protein concentration was determined with a Pierce™ BCA protein assay (Thermo Fisher Scientific; Cat# 23225), and lysates were stored at −80°C until used. Proteins were denatured (100°C for 5 min in a dry-bath incubator) in NuPage LDS sample buffer (Thermo Fisher Scientific; Cat# NP0007) with 5% 2-β-mercaptoethanol. Samples were subjected to SDS-PAGE on 8% or 12% acrylamide gels at 10 μg protein/lane, and electrophoresed for 1.5–2 h at 80 mV (stacking gel) and 120 mV (resolving gel). Proteins were then transferred to a PVDF membrane and blocked (2–3 h) in 5% non-fat dry milk in Tris-Tween buffered saline (TTBS).

Protein levels were measured for IFNGR1, TGFβR1, CD206 and KCa3.1. Polyclonal rabbit primary antibodies were applied overnight (4°C) in TTBS with 1% BSA, as follows: anti-IFNGR1 (1:250, Biorbyt, San Francisco, CA, RRID:SCR_013542; Cat# orb338837), anti-TGFβR1 (1:500; Abcam; Cat# ab31013, RRID:AB_778352), anti-CD206 (1:2000, Abcam; Cat# ab64693, RRID:AB_1523910), and anti-KCNN4 (KCa3.1; 1:2000, Bioss Inc., Boston, MA, USA; Cat# bs-6675R, RRID:AB_11058017). After washing in 1% BSA-TTBS (4 × 10 min), membranes were incubated for 1 h at room temperature in horseradish peroxidase-labeled secondary anti-rabbit IgG antibodies (1:3000; Cedarlane, Burlington, ON, Canada, RRID:SCR_004462; Cat# CLAS09-602) in 1% BSA-TTBS. After repeated washes, membranes were treated with GE Healthcare ECL™ Start Western Blotting Detection Reagent (Sigma-Aldrich; Cat# GERPN3243), and protein bands were visualized using the ChemiDoc™ XRS System (Bio-Rad).

We used total protein normalization of Coomassie-stained immunoblots (Welinder and Ekblad, 2011) by adding 0.1% Coomassie Brilliant Blue G (Sigma-Aldrich; Cat# B8522) for 1 min, de-staining for 2 min in acetic acid/methanol/water (1:5:4), air-drying, and imaging with a ChemiDoc™ XRS System. Using Image Lab (ver.5.2.1, RRID:SCR_014210), gel lanes and bands were user-delineated, background was subtracted using the software’s rolling disk algorithm, and signal intensities of identified bands were determined. Bands of interest were then normalized to the total Coomassie blue staining intensity of a given lane, and expressed as fold-changes relative to unstimulated (control) cells. Supplementary Figure S1 shows uncropped images of representative blots used for quantification.

### Whole-Cell Patch-Clamp Recordings

Microglia were plated at 7–9 × 10^4^ cells/coverslip, mounted in a 300 μL volume perfusion chamber (Model RC-25, Warner Instruments, Hamden, CT, USA) at room temperature, and recorded in the whole-cell configuration. The bath solution consisted of (in mM): 125 NaCl, 5 KCl, 1 CaCl_2_, 1 MgCl_2_, 10 HEPES, 5 D-glucose, adjusted to pH 7.4 and 290–300 mOsm/kg H_2_O. Pipettes were pulled from thin wall borosilicate glass (World Precision Instruments, Sarasota, FL, USA, RRID:SCR_008593) on a Narishige puller (Narishige Scientific, Setagaya-Ku, Tokyo) and fire polished with a microforge (MF 900; Narishige) from about 2 MΩ to 5–8 MΩ. The pipette (internal) solution was 40 KCl, 100 K aspartate, 1 MgCl_2_, 10 HEPES, 2 MgATP (pH 7.2; 290–300 mOsm/kg H_2_O), and with 0.5 CaCl_2_ and 1 EGTA to buffer internal free Ca^2+^ to ~120 nM. The same bath and pipette solutions were used to record all currents. Data were acquired using an Axopatch 200A amplifier, filtered at 5 Hz with a DigiDATA 1322A board, and analyzed with pCLAMP 10 software (all from Molecular Devices, Sunnyvale, CA, USA). Junction/offset potentials were calculated with the pCLAMP utility and used to correct each figure. Voltage protocols are indicated in the Figure Legends and the rationale briefly explained in the “Results” section text.

#### Channel Blockers and Activators

To quantify the Kv1.3 current, the potent Kv1.3 blocker, agitoxin-2 (5 nM AgTx-2; Sigma-Aldrich, Cat# A9219) was added to the bath and the remaining current was subtracted from the total current to yield the Kv1.3 component. AgTx-2 was prepared from a stock solution in double-distilled water with 0.02% BSA. To quantify Kir2.1, the current was blocked with 20 μM ML133 (Tocris Bioscience, MO, RRID:SCR_003689; Cat# 4549) or Ba^2+^, and the blocker-sensitive Kir2.1 component was assessed, as before (Lam and Schlichter, 2015; Lam et al., 2017). Ba^2+^ was prepared in double-distilled water, and ML133 was prepared from a stock solution in dimethyl sulfoxide (DMSO; Tocris Bioscience; Cat# 3176). To quantify the KCa3.1 current, it was evoked by the positive-gating modulator, 1 μM SKA-111 (gift from Prof. Heiki Wulff, Department of Pharmacology, University of California, Davis, CA, USA; Wulff and Castle, 2010) followed by addition of the blocker, 1 μM TRAM-34 (prepared in DMSO; Sigma-Aldrich; Cat# T6700; Coleman et al., 2014), after which the blocker-sensitive KCa3.1 component was calculated.

### Statistics

Quantified data are expressed as mean ± SEM for the number of biological replicates indicated in figure and table legends. For Nanostring mRNA analysis, a one-way analysis of variance (ANOVA) with Dunnett’s *post hoc* test was used to assess TGFβ1-induced changes within a rodent species. To compare effects of TGFβ1 and IL-10 between rat and mouse microglia, mRNA counts were normalized to housekeeping genes, and then results were expressed as fold-changes relative to control cells. A 2-way ANOVA with Fisher’s LSD test was conducted in Graphpad Prism ver 6.01 (RRID:SCR_002798). The *p* values for differences in treatment or species were adjusted using a 5% false discovery rate correction for multiple comparisons (Benjamini and Yekutieli, 2001) in the program R (version 3.3.1; R Project for Statistical Computing, RRID:SCR_001905). Proliferation, apoptosis, Western blotting, and electrophysiological data were analyzed in GraphPad Prism using a 2-way ANOVA with Bonferroni or Tukey’s *post hoc* analysis.

## Results

**Figure 1:**
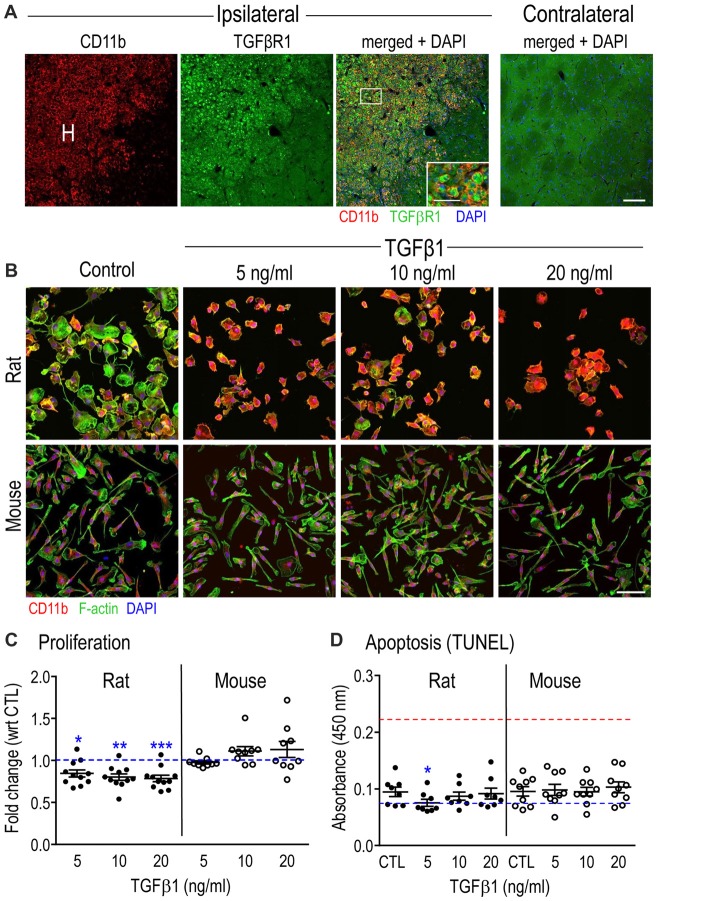
Transforming growth factor β1 (TGFβ1) receptor in microglia after Intracerebral hemorrhage (ICH), and general effects of TGFβ1 on microglia *in vitro*. **(A)** Microglia/macrophages increase in the hematoma (H) after ICH and express TGFβR1. Representative confocal micrographs taken at 7 days in the damaged ipsilateral rat striatum (left) and the undamaged contralateral striatum (right). The inset is a higher magnification image of the boxed area (scale bar, 20 μm) showing microglia and infiltrating macrophages in the hematoma labeled with CD11b (OX-42 antibody; red) and TGFβR1 (green). Cell nuclei are labeled with 4’-6-diamidino-2-phenylindole (DAPI; blue). Scale bar for main images is 100 μm. **(B)** The morphology of isolated microglia (stained for CD11b, red, as in **(A)** was assessed using phalloidin to stain F-actin (green) and DAPI (blue) to label nuclei. Images are representative of results from six separate cell cultures for each species. Scale bar is 100 μm. **(C)** Microglia proliferation was determined 24 h after TGFβ1 treatment using the CyQUANT™ assay. Results are shown as fold-change with respect to untreated (control, CTL) microglia (blue dashed line) and expressed as mean ± SEM for 9–11 individual cell cultures. **(D)** Apoptosis was monitored using the TiterTACS™ colorimetric assay at 24 h after TGFβ1 treatment. Results are shown as mean absorbance at 450 nm (mean ± SEM, 8–9 individual cultures). The blue dashed line indicates values for the negative controls, and the red dashed line indicates the positive controls. Significant differences are shown between control and TGFβ1 treated cells (*). One symbol of indicates *p* < 0.05; 2, *p* < 0.01; 3, *p* < 0.001.

As mentioned in the “Introduction” section, TGFβ1 is often increased after acute CNS damage, and microglia are important responders to this cytokine. We previously reported an increase in TGFβ1 mRNA in the damaged rat striatum after ICH was induced by collagenase injection (Lively and Schlichter, 2012). Before assessing TGFβ1 effects on isolated microglia, we wanted to ensure that the TGFβR1 receptor is well expressed on microglia in this model of acute CNS injury. We previously showed that at 3–7 days after ICH in the rat striatum, the number of microglia/activated macrophages dramatically increases in the hematoma (Wasserman and Schlichter, 2007; Wasserman et al., 2007; Moxon-Emre and Schlichter, 2011; Lively and Schlichter, 2012). Here, we show that at 7 days after ICH, there was substantial TGFβR1 staining in microglia/macrophages in the hematoma (Figure 1A; CD11b staining; the hematoma was readily identified by the presence of blood). More distant from the hematoma (toward the right side of each image), and in the undamaged contralateral striatum, there was ubiquitous, diffuse TGFβR1 staining. The remainder of this study addresses the questions: what are the effects of TGFβ1 exposure on isolated microglia, and do the outcomes differ between rat and mouse?

### Effects of TGFβ1 on Microglial Morphology, Proliferation and Apoptosis

To assess the general health of isolated rat and mouse microglia and their response to TGFβ1, we first examined morphology, proliferation and apoptosis in a pilot study using 5, 10 and 20 ng/ml of TGFβ1 (Figures 1B–D). As seen in Figure 1B, untreated cells had a similar morphology to our recent studies of primary rat (Siddiqui et al., 2012; Lively and Schlichter, 2013; Lam et al., 2017) and mouse (Lam et al., 2017) microglia. That is, about half the cells had a distinct unipolar, migratory phenotype with a lamellum at the leading edge and a tail/uropod at the trailing end (50 ± 2%, 12 individual cultures for rat; 53 ± 2%, eight individual cultures for mouse). For control cells, the main species differences are the wider lamellum in rat (32.5 ± 1.5 μm) than mouse (25.4 ± 0.9 μm; *p* = 0.0025, unpaired *t*-test), and greater cell length in mouse (79.2 ± 3.9 μm) than rat (68.0 ± 2.0 μm; *p* = 0.0273, unpaired *t*-test). Consequently, the aspect ratio (lamellum width ÷ cell length) was 0.54 ± 0.02 for rat and 0.35 ± 0.01 for mouse (*p* < 0.0001). Moreover, only in rat cells was the lamellum ever wider than the cell length.

TGFβ1-treated rat cells showed increased CD11b staining and retracted their lamellae and uropods at all concentrations tested. However, visual inspection cannot determine whether the rounding up reflects a change in size or just a change in morphology at a constant size. The measured membrane capacitance can be used as a direct measure of total cell membrane area, regardless of 3-dimensional cell shape. For instance, capacitance accounts for retraction of cell processes, membrane ruffling and changes in cell height. This is because the bimolecular lipid membrane is a remarkably consistent dielectric, and specific membrane capacitance is, in essence, a biological constant (~1 μF/cm^2^). We measured cell capacitance during patch-clamp experiments (below) and showed no differences between species or with TGFβ1 treatment. For control microglia, the values (mean ± SEM) were 25.3 ± 1.8 pF (*n* = 32) for rat, and 24.2 ± 1.4 pF (*n* = 45) for mouse. For mouse microglia, 24 h treatment with TGFβ1 did not obviously affect the morphology or intensity of CD11b staining, and the capacitance was 22.1 ± 1.0 pF (*n* = 41; *p* = 0.2206, unpaired *t*-test). Despite rat cells rounding up, their capacitance was not affected (22.3 ± 1.5 pF; *n* = 29; *p* = 0.2125, unpaired *t*-test). Thus, the shape changes were due to rounding up, not to cell size changes.

The longest TGFβ1 treatment time used for gene and protein analysis in this study was 24 h. The proliferation assay showed a modest decrease (20%–25%) at 24 h in the number of rat microglia at all TGFβ1 concentrations; while mouse cell numbers were unaffected (Figure 1C). Despite the decrease in rat cell numbers, TGFβ1 treatment did not evoke apoptosis at 24 h in either species at any concentration tested (Figure 1D). This suggests that the decrease in rat cell numbers was an effect on cell cycle, although this was not directly examined. Based on the morphological changes and slightly decreased proliferation of rat microglia, we chose the lowest effective TGFβ1 concentration (5 ng/ml) for subsequent rat experiments, while 20 ng/ml was used for mouse.

### Transcriptional Profiling to Compare Rat and Mouse Microglial Response to TGFβ1

The next goal was to compare responses of rat and mouse microglia to TGFβ1 by profiling transcript expression of >50 selected genes. We have organized the results into functional groupings, including microglial markers and immunomodulators, pro- and anti-inflammatory mediators and their receptors, and some molecules related to specific microglial physiological functions. The gene nomenclature is indicated for rat (Supplementary Table S1) and mouse (Supplementary Table S2), and where different, the rat names are used.

#### Microglia Markers and Immune Modulators

First, we quantified expression of several genes that are often used to identify microglial “activation” after acute brain injury, as well as several immunomodulatory molecules (Table 1; Supplementary Table S3).

**Table 1:** Transcript expression of microglia markers and immune modulators.

	Control		TGFβ1	
	*Relative mRNA counts ± SD*		*Fold change wrt control*	
Gene	Rat	Mouse	Rat	Mouse
*Csf1r*	29118 ± 2166***	5299 ± 880	1.29^↑↑^**	1.01
*Cx3cr1*	1033 ± 238	3112 ± 746***	13.23^↑↑^**	2.07^↑↑^
*Itgam (CD11b)*	6527 ± 1650**	3054 ± 586	2.25^↑↑^**	1.17
*Nr3c1 (GR)*	1481 ± 170***	457 ± 41	1.06	0.76^↓↓^**
*Socs1*	19 ± 7	12 ± 8	0.39^↓^	1.10
*Tlr4*	694 ± 119	700 ± 50	1.09	0.59^↓↓^**

*Rat and mouse microglia were stimulated for 24 h with transforming growth factor β1 (TGFβ1). To assess species differences in basal expression, unstimulated (control) mRNA counts/200 ng RNA sample are expressed as mean ± SEM (*n* = 4–6 individual cultures). For clarity, protein names are included for some genes. Effects of TGFβ1 on a given gene are expressed as fold changes with respect to species-matched control levels. Arrows indicate statistically significant increases (↑) or decreases (↓) in expression within a species. Species differences are indicated by bold numbers and asterisks. One symbol (arrow or asterisk) indicates *p* < 0.05; two, *p* < 0.01; three, *p* < 0.001*.

##### Control

Microglia of both species expressed moderate levels (>100 counts) of several well-known receptors on microglia: *Itgam* (CD11b), *Cx3cr1* (fractalkine receptor), toll-like receptors 2 and 4 (*Tlr2*, *Tlr4*) and *Nr3c1* (glucocorticoid receptor, GR), and high levels (>5000 counts) of *Cd68* (ED1) and colony-stimulating factor receptor (*Csf1r*; c-fms). The most striking species differences in control cells were higher *Cd68* and much higher *Aif1* (Iba1) in rat, as recently reported (Lam et al., 2017). Among known immune modulators, both species expressed low levels (<100 counts) of suppressor of cytokine signaling 1 (*Socs1*) and *Socs*3, and moderate levels of *Nfkbia* (IκBα; endogenous inhibitor of NFκB) and translocator protein (*Tspo*).

##### TGFβ1 Treated

Of genes in this category, 6/12 were altered in one or both species but there were several species differences in the magnitude of responses (Table 1). For instance, rat microglia had greater up-regulation of *Cx3cr1*, *Itgam* and *Csf1r*; mouse cells had reduced *Nr3c1* and *Tlr4* ; and rat cells had reduced *Socs1*. Several genes in this category were not significantly altered in either species: *Aif1*, *Cd68*, *Nfkbia*, *Socs3*, *Tlr2* and *Tspo* (Supplementary Table S3). There were several similarities in a pilot study of 6 h TGFβ1 treatment of rat cells, including up-regulated *Cx3cr1* and *Itgam* but no changes in *Tlr2* or *Tlr4* (not illustrated).

#### Anti-inflammatory Genes and Receptors

##### Control

Microglia of both species had moderate resting mRNA levels of *Tgfb1*, and its cognate receptors, *Tgfbr1* and *Tgfbr2*; however, rat cells had higher *Tgfb1* and *Tgfbr1* (Table 2). Both species had low resting levels of the interleukins, *Il4* and *Il10*, but moderate levels of their receptors (*Il4r*, *Il13ra1*, *Il10ra*, *Il10rb*). Several genes associated with alternative activation were very low in both species: *Arg1*, *Ccl22*, *Cd163* and *Retnla* (FIZZ1). *Il1rn, Mrc1* (CD206), *Myc*, *Pparg* and *Trem2* were moderately expressed in both species. The main species differences were that unstimulated rat cells had higher levels of *Il1rn*, *Il4r, Il10ra*, *Il10rb*, *Pparg*, *Tgfb1*, *Tgfbr1* and *Trem2*.

**Table 2:** Transcript expression of anti-inflammatory genes and receptors.

	Control		TGFβ1	
	*Relative mRNA counts ± SD*		*Fold change wrt control*	
Gene	Rat	Mouse	Rat	Mouse
*Il1rn*	3942 ± 1620***	534 ± 228	0.45^↓^	0.50
*Il4*	10 ± 4	64 ± 9***	1.63	1.97^↑↑^
*IL4r*	595 ± 31***	162 ± 60	1.02	2.28^↑↑^**
*Il10*	12 ± 12	24 ± 2	0.18	2.28^↑↑^**
*Il13ra1*	543 ± 75	413 ± 72	0.84	1.38^↑↑^**
*Mrc1*	1869 ± 899	1779 ± 954	0.07^↓↓^	0.11^↓↓^
*Myc*	650 ± 90	490 ± 88	2.01^↑↑^**	0.44^↓↓^
*Pparg*	833 ± 346***	111 ± 16	0.04^↓↓^	0.35^↓↓^
*Tgfb1*	16451 ± 1082***	2964 ± 259	1.41^↑↑^**	0.72^↓^
*Tgfbr1*	3811 ± 530***	1156 ± 213	8.07^↑↑^**	2.57^↑↑^
*Tgfbr2*	1179 ± 90	1295 ± 211	1.99^↑↑^**	1.37
*Trem2*	6255 ± 882***	225 ± 12	1.40^↑↑^**	1.03

*Treatments, data presentation and analysis were as in Table 1*.

##### TGFβ1 Treated

**Figure 2:**
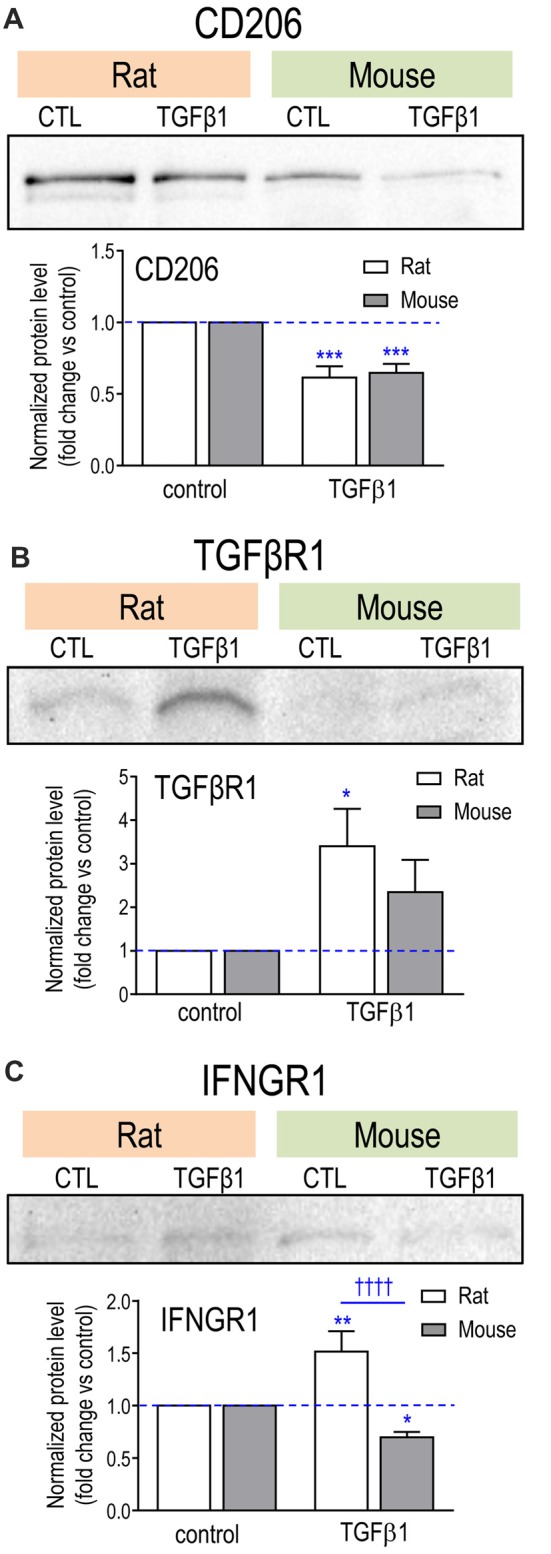
Effect of TGFβ1 treatment on protein levels for exemplary molecules.** (A)** CD206;** (B)** TGFβR1; **(C)** IFNGR1. Microglia were unstimulated (CTL, control) or stimulated with TGFβ1 for 24 h. Representative Western blots, with both species on the same gel. For each blot, the band of interest was normalized to total protein in that lane (Coomassie blue staining), and then the fold-changes were calculated with respect to unstimulated (control) microglia (mean ± SEM; 4–7 individual cultures for mouse, 5–6 for rat). Significant differences are shown between control and TGFβ1 treated cells (*), and between species (†). One symbol of either type indicates *p* < 0.05; 2, *p* < 0.01; 3, *p* < 0.001; and 4, *p* < 0.0001.

Changes were seen in 12/18 genes examined in one or both species. Both species had decreased *Mrc1* and *Pparg*, and increased *Tgfbr1* (Table 2). We previously showed that mRNA changes in several hallmark genes corresponded well with protein changes in both rat and mouse microglia (Lam et al., 2017). Here, we corroborated protein changes for two exemplary molecules. Figure 2 shows representative Western blots and summarized protein changes for CD206 (*Mrc1*; Figure 2A) and TGFβR1 (Figure 2B). In accord with the mRNA changes, CD206 protein decreased in both species (Figure 2A), and TGFβR1 protein increased, particularly in rat microglia (Figure 2B). With respect to mRNA levels, there were several species differences in the magnitude or even in the direction of responses. For instance, *Tgfb1* and *Myc* increased in rat but decreased in mouse. Only rat cells showed increases in *Tgfbr2* and *Trem2*, and only mouse cells showed increases in *Il4*, *Il10*, *Il4r* and *Il13ra1*. Interestingly, neither species showed a change in several alternative activation-associated genes: *Arg1*, *Ccl22*, *Cd163*, *Retnla* or the IL10 receptors: *Il10ra and Il10rb* (Supplementary Table S3). A pilot study of 6 h TGFβ1 treatment of rat cells showed several similarities: decreased *Mrc1* and *Pparg*, and no change in *Ccl22* (not illustrated).

#### Pro-inflammatory Genes and Receptors

##### Control

As we previously found for rat microglia (Sivagnanam et al., 2010; Liu et al., 2013; Lively and Schlichter, 2013; Siddiqui et al., 2016; Lam et al., 2017); here, unstimulated microglia from both species were in a relatively resting state, exemplified by very low expression of several pro-inflammatory mediators: *Nos2* (iNOS), *Il6*, *Ptgs2* (COX-2), *Ifng* and *Il1r1* (IL-1β receptor; Supplementary Table S3). Nevertheless, cells of both species were poised to respond to the pro-inflammatory cytokines, IFNγ and TNFα, having moderate levels of the receptors, *Ifngr1*, *Tnfrsf1a* (TNFR1) and *Tnfrsf1b* (TNFR2; Table 3; Supplementary Table S3). Both species also expressed moderate levels of *Tnf* (TNFα, which is normally expressed in the healthy brain), caspase-1 (*Casp1*), IL-1-converting enzyme (ICE), *Ccl3* (macrophage inflammatory protein 1-alpha), and the protein kinase, *Ptk2b* (proline-rich tyrosine kinase 2, PYK2). The main species differences were that rat showed much lower *Ifngr2* and higher *Ccr5*, *Ifngr1*, *Il1b, Tnfrsf1a, Tnfrsf1b* and *Trem1*.

**Table 3:** Transcript expression of pro-inflammatory genes and receptors.

	Control		TGFβ1	
	*Relative mRNA counts ± SD*		*Fold change wrt control*	
Gene	Rat	Mouse	Rat	Mouse
*Casp1*	768 ± 179	937 ± 126	0.89	0.49^↓↓^**
*Ccl3*	5811 ± 1393	4626 ± 2083	0.26^↓↓^	0.30^↓^
*Ccr5*	3357 ± 364***	412 ± 187	0.37^↓↓^	0.24^↓↓^
*Ifngr1*	7723 ± 1111***	2654 ± 259	2.12^↑↑^**	0.50^↓↓^
*Il1b*	1475 ± 993***	52 ± 29	0.10^↓↓^	0.54
*Tnfa*	493 ± 213	242 ± 142	0.25^↓↓^	0.63
*Tnfrsf1b*	1839 ± 74***	676 ± 92	0.39^↓↓^*	1.57
*Trem1*	212 ± 162**	12 ± 5	3.45^↑^	4.05^↑↑^

*Treatments, data presentation and analysis were as in Table 1*.

##### TGFβ1 Treated

There were changes in 8/17 in one or both species, but again, there were species differences. *Ifngr1* increased in rat but decreased in mouse. Representative Western blots and summarized protein changes for IFNGR1 (Figure 2C) corroborate this species difference in response to TGFβ1 treatment. In rat, five genes were decreased (*Ccl3*, *Ccr5*, *Il1b*, *Tnfa*, *Tnfrsf1b*) and *Trem1* was increased. There were some similarities in mouse (decreased *Ccl3* and *Ccr5*, increased *Trem1*) but a unique response was decreased *Casp1*, an enzyme that contributes to IL1β activation. Hallmark pro-inflammatory genes that did not change in either species included* Ccr2, Ifng*, *Ifngr2, Il1r1, Il6*, *Nos2*, *Ptgs2*, *Ptk2b* and *Tnfrs1a* (Supplementary Table S3). It appears that many transcriptional responses to TGFβ1 were rapid; i.e., in a pilot study of 6 h treatment of rat microglia (not illustrated), 17/25 genes showed qualitatively similar responses to those seen at 24 h; e.g., increases in *Cx3cr1*, *Itgam*, *Ncf1* and *P2rx7;* decreases in *Tnfa*, *Mrc1* and *Pparg*, and no changes in* Nos2*, *Il4*, *Il4ra*, *Tlr4*, *Cd163* and *Retnla*.

#### Additional Genes Related to Microglial Physiology

Our earlier qRT-PCR (Sivagnanam et al., 2010) and recent Nanostring analyses (Siddiqui et al., 2016; Lam et al., 2017) showed that unstimulated rat microglia express several phagocytosis-related receptors, oxygen radical-producing NOX enzymes, as well as purinergic receptors that can respond to adenosine triphosphate (ATP) released from damaged and dying cells, modulate microglial phagocytosis, reactive oxygen species production and migration. Expression of some of these molecules changed according to the activation state (Sivagnanam et al., 2010; Siddiqui et al., 2016; Lam et al., 2017). For instance, those studies showed that pro-inflammatory stimuli elevated *Cybb* (NOX2)*, Ncf1, Fcgr3a*; while IL-4 treatment reduced *Cybb* and increased *Fcgr2b*. *Cybb* is the main contributor to the respiratory burst in microglia (Siddiqui et al., 2016), and its activation requires (neutrophil cytosolic factor 1, *Ncf1*/p47phox) relocation to the plasma membrane (reviewed in El-Benna et al., 2009). The voltage-gated proton channel, *Hvcn1* (Hv1), can promote NOX enzyme activity by allowing H^+^ efflux as charge compensation for NADPH-generated electrons (Ramsey et al., 2009; DeCoursey, 2013).

##### Control

Unstimulated microglia from both species had very low expression of *Nox1*, *Nox4*, *P2ry2*, *Adora1* (adenosine A_1_ receptor), and *Adora2a* (adenosine A_2A_ receptor). Both species had moderate to high levels of *Hvcn1*, *Cybb*, *Ncf1*, *P2rx7*, *P2ry12*, *Itgb2* and the phagocytosis-related molecules, *Fcgr1* (CD64), *Fcgr2b* (CD32), *Fcgr3a* (CD16) and *Msr1* (SR-A; Table 4; Supplementary Table S3). The main species differences were that rat cells had higher levels of *Hvcn1*, *Ncf1*, *Fcgr1*, *Fcgr2b, Itgb2*, *P2ry12* and *Msr1*, while mouse had higher levels of only *Cybb*.

**Table 4:** Transcript expression of additional genes related to microglial physiology and ion signaling.

	Control		TGFβ1	
	*Relative mRNA counts ± SD*		*Fold change wrt control*	
Gene	Rat	Mouse	Rat	Mouse
*Fcgr2b*	5614 ± 1350*	3306 ± 581	1.45^↑^	2.04^↑↑^
*Fcgr3a*	8039 ± 4607	4170 ± 1069	0.18^↓↓^**	0.68
*Itgb2*	8453 ± 744***	4209 ± 533	1.27^↑^	1.22
*Ncf1*	8223 ± 2318***	1016 ± 89	2.76^↑↑^**	1.89^↑↑^
*Nox1*	13 ± 6	11 ± 3	3.52^↑↑^	2.06^↑^
*Orai3*	437 ± 27***	127 ± 12	1.35^↑↑^	1.19
*P2rx7*	182 ± 41	240 ± 33	2.56^↑↑^**	0.97
*P2ry12*	431 ± 48**	146 ± 66	1.55^↑↑^	1.04
*Ptpn6*	2497 ± 418	2633 ± 351	0.99	0.68 ↓**
*Rest*	922 ± 82***	427 ± 15	1.25^↑↑^	1.16^↑^
*Trpm7*	695 ± 38**	459 ± 29	1.17^↑↑^**	0.91

*Treatments, data presentation and analysis are as in Table 1*.

##### TGFβ1 Treated

Several genes were affected by TGFβ1 (Table 4), including up-regulated *Fcgr2b*, *Ncf1* (higher in rat) and *Nox1* in both species. Species differences were decreased *Fcgr3a* and increased *P2rx7* (also seen at 6 h, not illustrated) and *P2ry12* in rat cells only. In both species, *Adora1, Adora2a, Cybb, Fcgr1*, *Hvcn1, Msr1*, *Nox4* and *P2ry2* were unchanged (Supplementary Table S3).

### Ion Channels and Regulators

Many microglial functions depend on Ca^2+^ signaling (see “Introduction” and “Discussion” sections); thus, we asked whether TGFβ1 treatment affects expression of several ion channel genes that are linked to Ca^2+^ entry. These included the Ca^2+^-permeable channels, *Trpm2*, *Trpm4* and *Trpm7*, the pore-forming subunits of CRAC channels (*Orai1*, *Orai3*), and the CRAC regulator, *Stim1*. We then assessed several K^+^ channels that potentially regulate the driving force for Ca^2+^ entry: *Kcna2* (Kv1.2), *Kcna3* (Kv1.3), *Kcna5* (Kv1.5), *Kcnj2* (Kir2.1), *Kcnma1* (KCa1.1/BK/maxi K), *Kcnn3* (KCa2.3/SK3) and *Kcnn4* (KCa3.1/SK4). In addition, several important K^+^ channel regulators were monitored: *Ptpn6* (Src homology region 2 domain-containing phosphatase-1; SHP-1), calmodulin (*Calm*), RE1 silencing transcription factor (*Rest*), *Phtp1* (phosphohistidine phosphatase 1, PHP), and *Nme2* (nucleoside diphosphate kinase 2, NDPK2). Note: for ease of comparison with the literature, results are presented using the protein names. While modest effects of TGFβ1 were seen in one or both species for Orai3, Rest, SHP-1 and Trpm7 (Table 4), several genes were unaffected: Kv1.2, Kir2.1, KCa1.1, Trpm2, Trpm4, Orai1, Stim1, and the KCa channel regulators, calmodulin, NDPK2 and PHP (Supplementary Table S3). Some of these were not considered further. We then focussed on the K^+^ channels, Kv1.3, Kir2.1 and KCa3.1, quantifying both mRNA expression and ion currents (the ultimate output of gene and protein expression). Those results are organized below under each channel type. For Kv1.3 and Kir2.1, adult microglia were also examined because some researchers have questioned whether their expression differs from neonatal microglia (see “Discussion” section).

### TGFβ1 Increases Kv1.3 mRNA and Current in Neonatal and Adult Rat and Mouse Microglia

**Figure 3:**
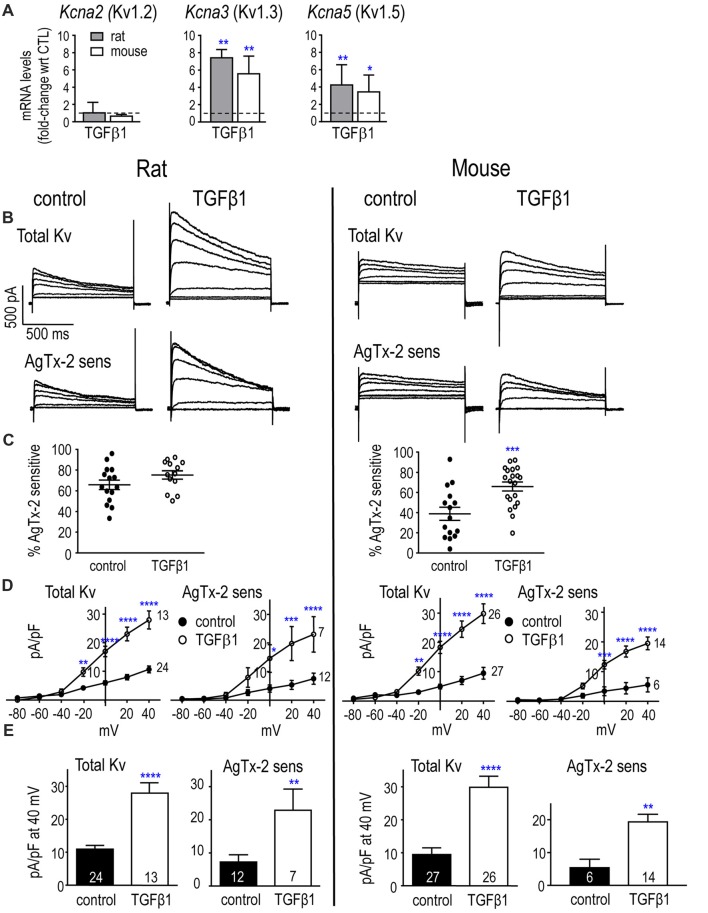
In neonatal rat and mouse microglia, TGFβ1 increases *Kcna3* mRNA expression and Kv1.3 current.** (A)** Transcript expression of selected Kv1-family channel members. Rat and mouse microglia were unstimulated (control) or stimulated with TGFβ1 for 24 h (expression data) or 30 h (whole-cell recordings). mRNA expression (mRNA counts/200 ng total RNA) was determined by NanoString and expressed as fold-change relative to unstimulated control cells (mean ± SEM; *n* = 4–6 individual cultures). **(B)** Representative whole-cell Kv currents in control (untreated) microglia, and in TGFβ1-treated cells. The voltage clamp protocol consisted of 1 s-long voltage steps from −80 mV to +40 mV in 20 mV increments, applied every 60 s from a holding potential of −100 mV. Then, for each cell, 5 nM AgTx-2 was perfused into the bath to record the AgTx-2-insensitive component, which was then subtracted from the total current to yield the Kv1.3 current. **(C)** Scatter plot of individual cells showing the proportion of the peak current (at +40 mV) that was blocked by AgTx-2. **(D)** Peak current density (pA/pF) as a function of voltage for the total Kv current and the AgTx-2-sensitive Kv1.3 component. Data are shown as mean ± SEM for the number of cells indicated. **(E)** Summary of current density (pA/pF) measured at +40 mV. For all graphs, one symbol indicates *p* < 0.05; 2, *p* < 0.01; 3, *p* < 0.001; and 4, *p* < 0.0001.

Transcripts and protein have been detected for Kv1.2, Kv1.3 and Kv1.5 in primary neonatal rat microglia (Kotecha and Schlichter, 1999; Khanna et al., 2001; Fordyce et al., 2005; Li et al., 2008), and primary neonatal mouse microglia (Kv1.2: Lam et al., 2017; Kv1.3: Schilling et al., 2000; Pannasch et al., 2006; Lam et al., 2017; Kv1.5: Pannasch et al., 2006). Here, unstimulated (control) microglia of both species expressed low transcript levels of Kv1.2 and Kv1.3 and nearly undetectable Kv1.5. mRNA counts/200 ng RNA were: *Kcna2* (Kv1.2) 73 ± 47 in rat, 10 ± 6 in mouse (*p* = 0.011; *n* = 6 each); *Kcna3* (Kv1.3) 109 ± 18 in rat, 70 ± 14 in mouse (*p* = 0.013); *Kcna5* (Kv1.5) 4 ± 2 in rat, 3 ± 2 in mouse. TGFβ1 treatment did not alter Kv1.2 mRNA, but significantly increased Kv1.3 mRNA: about 7.4-fold at 24 h in rat (~10 fold at 6 h, data not shown), and 5.6-fold in mouse (Figure 3A). Kv1.5 mRNA also increased about 4.2-fold in rat and 3.4-fold in mouse cells.

Low levels of mRNA for K^+^ channels can nonetheless result in robust currents, as we previously observed for Kv1.3 and Kir2.1 (Lam and Schlichter, 2015; Lam et al., 2017), possibly because channel proteins can have very slow turnover (Jugloff et al., 2000; Balut et al., 2012). Thus, it is important to measure the currents as a direct measure of functional ion channels. We used whole-cell patch-clamp recordings to compare the total Kv and Kv1.3 currents with and without TGFβ1 treatment. Cells were treated for 30 h to provide additional time for channel protein expression, trafficking and post-translational modifications, as before (Lam et al., 2017). The cell capacitance (shown in the first section of the “Results”) was used to normalize each current to the cell size.

#### Kv and Kv1.3 Currents

The voltage protocols used to quantify Kv currents were essentially as in our earlier work (Kotecha and Schlichter, 1999; Cayabyab et al., 2000; Newell and Schlichter, 2005; Lam et al., 2017). Importantly, they were designed to consider that the current amplitude depends on the holding potential, test potential, and frequency of depolarization (Grissmer et al., 1994; Nörenberg et al., 1994; Kotecha and Schlichter, 1999; Cayabyab et al., 2000). The holding potential (−105 mV) was used to relieve channel inactivation, and 60 s intervals between successive depolarizing steps allowed recovery from use-dependent (cumulative) inactivation. The rat protocol required ~20 min, and because mouse recordings did not usually last long enough, the protocol was modified to a voltage ramp from −75 mV to +45 mV from the −105 mV holding potential. This protocol was first validated by ensuring that the amplitude at +45 mV was the same as for a single voltage step.

##### Neonatal Rat

Kv currents were observed in every rat microglial cell examined, with or without TGFβ1 treatment (examples shown in Figure 3B), and a similar proportion of the total current was Kv1.3 (i.e., blocked by AgTx-2) in both groups: 66 and 75% (Figure 3C). Both the total Kv current and AgTx-2 sensitive Kv1.3 component activated in a time- and voltage-dependent manner above about −40 mV (corrected for junction potential offsets), and the currents increased with depolarization (Figures 3B,D). Time-dependent inactivation was also apparent during the 1-s depolarizing test pulses. TGFβ1 treatment increased the total Kv current and AgTx-2 sensitive Kv1.3 component (Figures 3D,E), and is consistent with the increase in Kv1.3 mRNA (Figure 3A).

##### Neonatal Mouse

Two species differences in control mouse microglia were the lower prevalence of observing a Kv current (18/31 cells) and that, among cells with a detectable current, less was AgTx-2-sensitive compared to control rat microglia (~40%; Figure 3C). This implies that unstimulated mouse microglia have an additional, unidentified Kv current type. Of note, all TGFβ1-treated mouse cells expressed a Kv current (20/20), and a greater proportion of the current was Kv1.3 (65%; *p* < 0.001). TGFβ1 increased the amplitudes of total Kv and Kv1.3 currents (Figures 3B,D), consistent with the increase in Kv1.3 mRNA. In both species, control and TGFβ1-treated cells produced substantial currents despite the relatively low *Kcna3* mRNA levels. This is consistent with our previous studies on neonatal microglia from rat (Kotecha and Schlichter, 1999; Cayabyab et al., 2000; Newell and Schlichter, 2005; Lam et al., 2017) and mouse (Lam et al., 2017).

##### Adult Rat and Mouse

**Figure 4:**
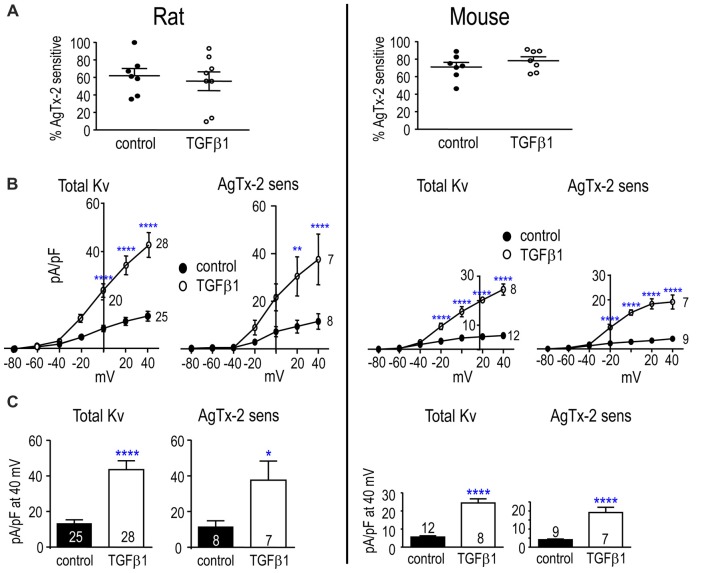
TGFβ1 increases the Kv1.3 current in adult rat and mouse microglia. Treatments and recording conditions were as in Figure 3. **(A)** Scatter plot of individual cells showing the proportion of the peak current (at +40 mV) that was blocked by AgTx-2. **(B)** Peak current density (pA/pF) as a function of voltage for the total Kv current and the AgTx-2-sensitive Kv1.3 component. **(C)** Summary of current density (pA/pF) measured at +40 mV. Data are shown as mean ± SEM for the number of cells indicated. One symbol of either type indicates *p* < 0.05; 2, *p* < 0.01; and 4, *p* < 0.0001.

Figure 4 shows Kv currents in adult rat and mouse microglia. In both species, a Kv current was always observed in untreated and TGFβ1-treated cells (>100 cells). In control cells, the proportion of Kv current contributed by Kv1.3 was similar in rat and mouse cells (~62 and 71%, respectively; Figure 4A). After TGFβ1 treatment, the Kv1.3 component was 78% in mouse but only 56% in rat because two cells appeared to express an AgTx-2-insensitive Kv current (Figure 4A). Effects of TGFβ1 on current amplitudes were similar to neonatal cells; i.e., both total Kv and Kv1.3 currents increased (Figures 4B,C).

### Kir2.1 mRNA and Current Were Unchanged By TGFβ1 in Neonatal and Adult Rat and Mouse Microglia

**Figure 5:**
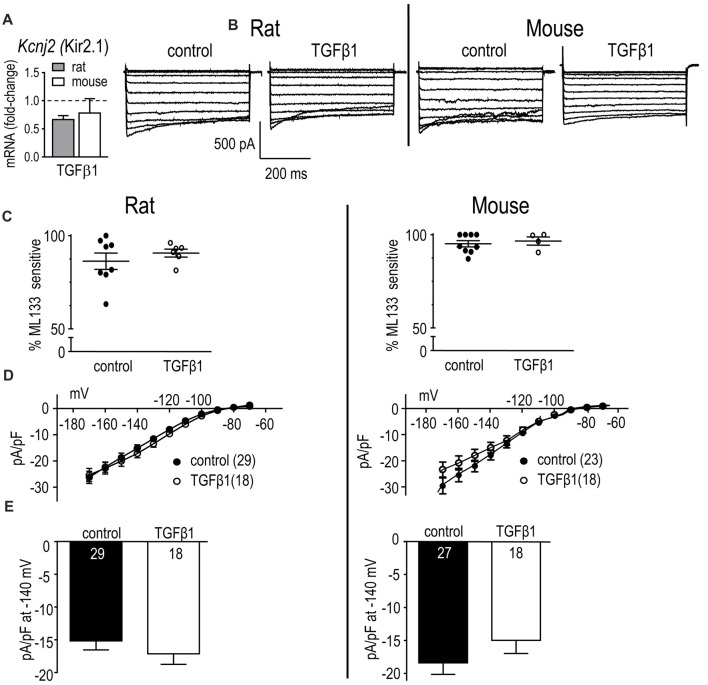
In neonatal rat and mouse microglia, TGFβ1 did not affect *Kcnj2* mRNA expression or Kir2.1 current. Microglia were unstimulated (control) or treated with TGFβ1 for 24 h (expression data) or 30 h (whole-cell recordings). **(A)** mRNA expression. Data are expressed as fold-change relative to control (unstimulated) microglia (mean ± SEM of 4–6 individual cultures). **(B)** Inward-rectifier (Kir) current. Representative traces of total inward current in control microglia, and in TGFβ1-treated cells. Whole-cell currents were recorded in response to 500 ms-long voltage steps between −170 mV and −70 mV in 10 mV increments from a holding potential of −20 mV. **(C)** Scatterplot of individual cells showing the proportion of the peak current (at −140 mV) that was blocked by 20 μM ML133. **(D)** Peak Kir2.1 current density (pA/pF) as a function of voltage. Data are shown as mean ± SEM for the number of cells indicated. **(E)** Summary of peak inward current density (pA/pF) measured at −140 mV. There were no significant TGFβ1 effects at the *p* < 0.05 confidence level.

Primary neonatal rat and mouse microglia express *Kcnj2* mRNA and Kir2.1 protein, and our recent studies strongly suggest that homomeric Kir2.1 channels produce the inward-rectifying K^+^ current in both species (Lam and Schlichter, 2015; Lam et al., 2017). In unstimulated microglia, there was a species difference with 4.5-fold higher *Kcnj2* mRNA expression in rat; i.e., mRNA counts/200 ng RNA were 1913 ± 657 in rat and 422 ± 90 in mouse (*p* < 0.001). TGFβ1 treatment did not significantly alter *Kcnj2* mRNA expression in either species (Figure 5A).

#### Kir Current

Inward currents were recorded in response to 500 ms-long voltage steps between −170 mV and −70 mV in 10 mV increments from a holding potential of −20 mV. A Kir current was detected in every rat and mouse microglial cell examined with and without TGFβ1 treatment. As previously described (Lam and Schlichter, 2015; Lam et al., 2017), microglia Kir currents display the stereotypical features of Kir2.1, with rapid activation at negative potentials, and current relaxation at very negative potentials (Figure 5B). The blocker, ML133 was used to quantify the Kir2.1 component of the whole-cell current, as before (Lam and Schlichter, 2015; Lam et al., 2017). In both species, almost all of the Kir current was blocked by 20 μM ML133 in both control and TGFβ1 conditions (86% and 91% in rat, 95% and 96% in mouse, respectively; Figure 5C); therefore, we subsequently compared the total Kir current density. In both species, the I-V relations showed inward rectification and reversal at about −82 mV after junction potential correction (Figure 5D), which is close to the K^+^ Nernst potential in these solutions (−84 mV). In control microglia, there was a similar magnitude of Kir2.1 current in both species, and TGFβ1 treatment had no effect (Figures 5D,E).

**Figure 6:**
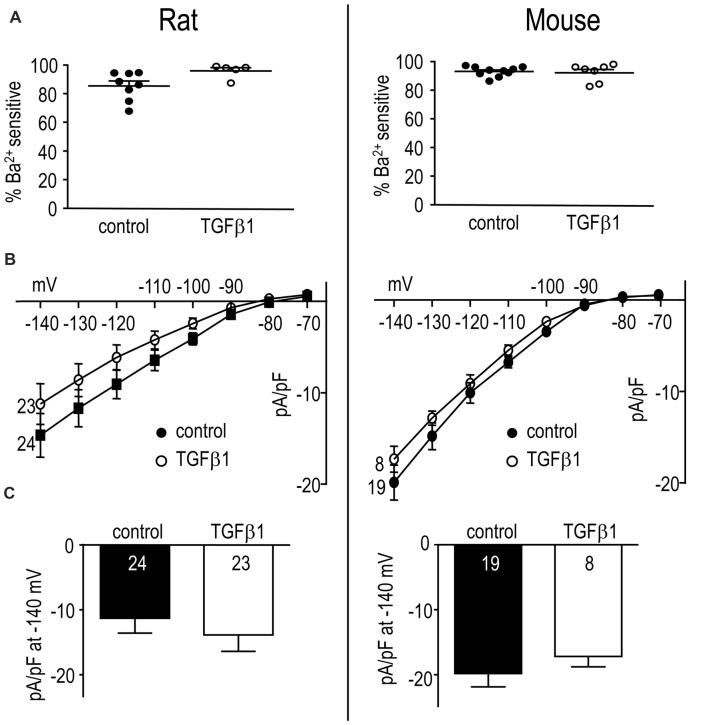
In adult rat and mouse microglia, TGFβ1 had no effect on the Kir2.1 current. Treatments and recording conditions were as in Figure 5. **(A)** Scatterplot of individual cells showing the proportion of the peak current (at −140 mV) that was blocked by 100 μM Ba^2+^. **(B)** Peak Kir2.1 current density (pA/pF) as a function of voltage (mean ± SEM for the number of cells indicated). **(C)** Summary of peak inward current density (pA/pF) measured at −140 mV. There were no significant TGFβ1 effects at the *p* < 0.05 confidence level.

Results from adult rat and mouse microglia are shown in Figure 6. In both species, Kir-like currents were always observed with or without TGFβ1 treatment (*n* > 70 cells), and almost all the current was contributed by Kir2.1 (i.e., blocked by Ba^2+^; Figure 6A). Similar to neonatal cells, TGFβ1 did not change the Kir2.1 current (Figures 6B,C).

### Species-Specific Effects of TGFβ1 on KCa3.1 mRNA and Current

**Figure 7:**
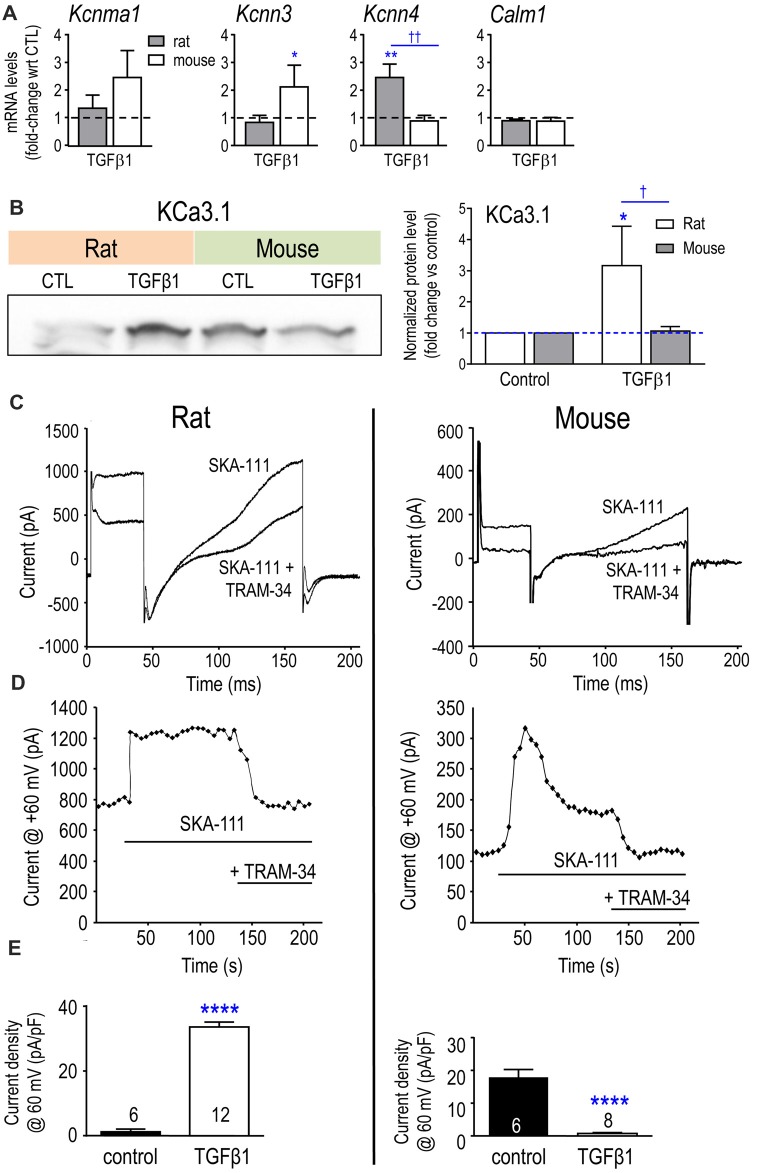
Expression of *Kcnn4* mRNA, KCa3.1 protein and current are differently affected by TGFβ1 in neonatal rat and mouse microglia. **(A)** Transcript expression of the Ca^2+^-activated K^+^ channels (mean ± SEM; 4–6 individual cultures): *Kcnma1* (KCa1.1/BK/maxi-K), *Kcnn3* (KCa2.3/SK3), *Kcnn4* (KCa3.1/SK4) and the regulatory molecule, calmodulin (*Calm1*). **(B)** Representative Western blot of KCa3.1 with both species on the same gel. Quantification resulting from normalization of each KCa3.1 band to total protein (Coomassie blue staining) in each lane. Fold changes were calculated with respect to unstimulated (control) microglia (mean ± SEM; 4–7 individual cultures for mouse, 5–6 for rat). For remaining figure, left panels represent neonatal rat microglia; right panels, neonatal mouse microglia. **(C)** Representative whole-cell recording showing KCa3.1 current evoked by the positive gating modulator, SKA-111 (1 μM), followed by inhibition by the blocker, 1 μM TRAM-34. From a holding potential of −90 mV, the voltage was stepped to +30 mV, followed by a ramp from −120 mV to +60 mV. **(D)** Examples of the time course, showing activation by the positive-gating modulator, SKA-111, and inhibition by the blocker, TRAM-34. **(E)** Summary of KCa3.1 current activation (i.e., the TRAM-34-sensitive component) in control cells vs. TGFβ1-treated microglia. Significant differences are shown between control and TGFβ1 treated cells (*), and between species (†). One symbol of either type indicates *p* < 0.05; 2, *p* < 0.01; and 4, *p* < 0.0001.

In control microglia, mRNA counts/200 ng RNA for the three K^+^ channels examined were:* Kcnma1* (KCa1.1/BK) 6 ± 3 in rat vs. 30 ± 10 in mouse (*p* < 0.01; *n* = 4–6 each); *Kcnn3* (KCa2.3) 25 ± 14 in rat vs. 9 ± 3 in mouse (*p* = 0.08); *Kcnn4* (KCa3.1) 52 ± 11 in rat vs. 644 ± 331 in mouse (*p* < 0.001). Calmodulin binds to KCa3.1 and KCa2-family channels and then, Ca^2+^ binding to calmodulin causes the channels to open (Xia et al., 1998; Fanger et al., 1999; Khanna et al., 1999; Wong and Schlichter, 2014). Of note, *Calm1* mRNA was 37-fold higher in unstimulated rat microglia (i.e., 21,304 ± 3538 counts in rat vs. 579 ± 40 in mouse, *p* < 0.001) and was not affected by TGFβ1 in either species (Figure 7A). A striking species difference was that TGFβ1 treatment increased *Kcnn3* in mouse cells only and increased *Kcnn4* in rat only (the *Kcnn4* result on rat was corroborated in a pilot study using quantitative RT-PCR, normalized to *Hprt1*, in which TGFβ1 up-regulated *Kcnn4* expression 4.5-fold (*p* < 0.01; *n* = 3; not illustrated). Furthermore, the species difference in *Kcnn4* mRNA corresponded with increased KCa3.1 protein in rat (but not mouse) microglia (Figure 7B).

KCa3.1 is the only Ca^2+^-activated K^+^ current we have detected in primary rat microglia (Khanna et al., 2001; Ferreira and Schlichter, 2013; Ferreira et al., 2014, 2015; Wong and Schlichter, 2014). To activate KCa3.1 currents during whole-cell recordings, we used a positive-gating modulator, either NS309 or SKA-111 (Coleman et al., 2014; reviewed in Balut et al., 2012). First, total inward and outward K^+^ currents were measured from a holding potential of −90 mV, by stepping to +30 mV followed by a ramp from −120 mV to +60 mV (Figure 7B). Then, TRAM-34 was added to the bath, and the KCa3.1 component was isolated by subtracting the unblocked current (almost entirely Kv at depolarized voltages). Figure 7C illustrates an example of the time course of experiments. Figure 7D shows that SKA-111 activated a very small KCa3.1 current in control rat microglia (1.2 ± 0.8 pA/pF); whereas, after TGFβ1 treatment, a large KCa3.1 current was activated in 12/19 cells (33.6 ± 1.5 pA/pF for responders; *p* < 0.0001). In a pilot study on rat microglia (not illustrated), 500 nM NS309 failed to activate a current (3/3 cells) but after TGFβ1 treatment there was a robust current in 6/8 cells (33.6 ± 5.3 pA/pF; *p* < 0.01). Surprisingly, the opposite was seen for mouse cells. SKA-111 activated a large KCa3.1 current in control mouse microglia (17.9 ± 2.4 pA/pF) but after TGFβ1 treatment, almost no current was evoked (0.8 ± 0.2 pA/pF; *p* < 0.0001).

### Comparing Microglial Responses to TGFβ1 and IL-10

**Figure 8:**
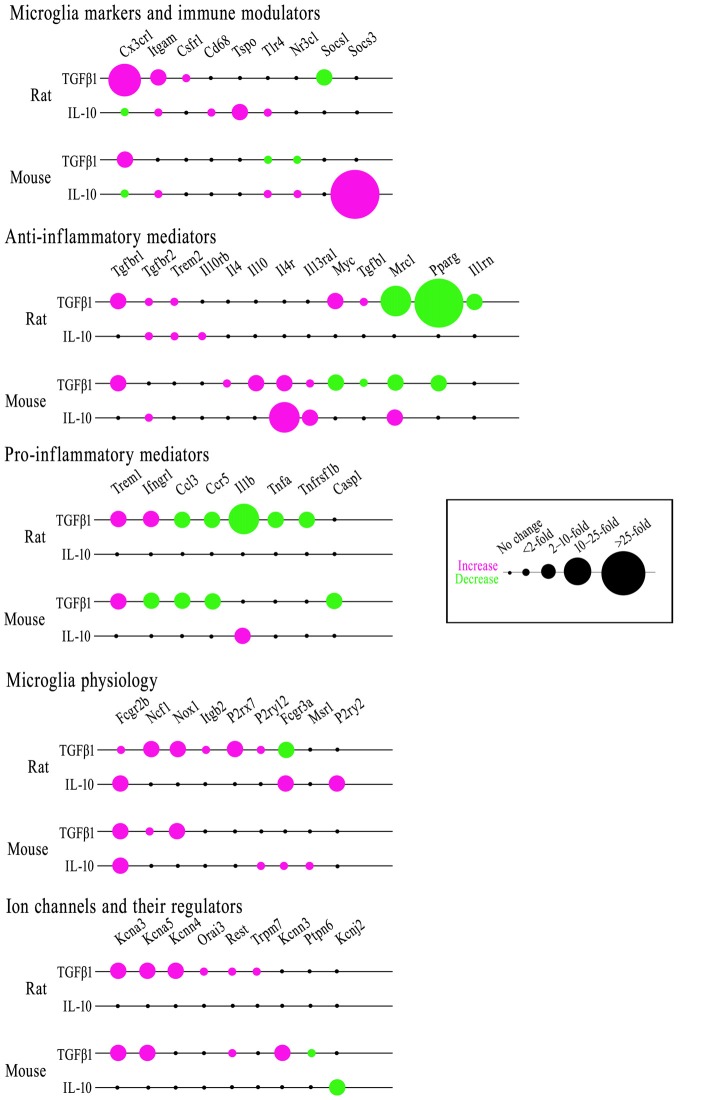
Comparing effects of TGFβ1 and IL-10 on gene expression in rat and mouse microglia. Microglia were stimulated with either cytokine for 24 h. mRNA transcript levels were determined by NanoString, expressed as fold-change relative to unstimulated control cells (*p* < 0.05 after FDR correction; *n* = 4–6 individual cultures). The bubble chart shows gene expression changes, sorted into bins according to ranges in the fold change. Magenta indicates increased expression; green shows decreases.

As anti-inflammatory or resolving cytokines, TGFβ1 and IL-10 are often assumed to have similar effects (see “Discussion” section). In Figure 8, we compare transcriptional responses of rat and mouse microglia to TGFβ1 (present study) with responses to IL-10 from our recent study (Lam et al., 2017) and some additional genes (*Ccl3*, *Ccr5*, *Csf1r*, *Itgb2*, *Kcnn3*, *Kcnn4*, *Orai3*). [Note: expression of 33/80 genes was unchanged; all the data are shown in Supplementary Table S4]. For ease of comparison, the bubble chart visually highlights both the magnitude and direction of changes. There were some intriguing differences in TGFβ1 and IL-10 responses, and we will highlight some examples under each category.

#### Microglial Markers and Immune Modulators

In both species, TGFβ1 increased *Cx3cr1* expression; whereas, IL-10 decreased it. TGFβ1 decreased *Socs1* only in rat. IL-10 increased *Socs3* only in mouse. IL-10 increased *Tlr4* in both species but TGFβ1 did not; indeed, *Tlr4* decreased in mouse. Both cytokines increased *Itgam* in rat; only IL-10 increased it in mouse. TGFβ1 increased *Csf1r* in rat only; IL-10 did not affect either species. IL-10 increased *Cd68* and *Tspo* in rat only. In mouse, TGFβ1 decreased *Nr3c1* but IL-10 increased it.

#### Anti-inflammatory Genes and Receptors

In both species, TGFβ1 decreased *Pparg* and increased *Tgfbr1*; both were unaffected by IL-10. TGFβ1 decreased *Mrc1* in both species; IL-10 increased it in mouse. TGFβ1 increased *Tgfb1* and *Myc* in rat but decreased them in mouse. However, TGFβ1 decreased *Il1rn* in rat. In mouse only, TGFβ1 increased *Il4* and *Il10* and both cytokines increased *Il4r* and *Il13ra1*. Both cytokines increased *Trem2* and *Tgfbr2* in rat; whereas, IL-10 only elevated *Tgfbr2* in mouse. IL-10 increased *Il10rb* only in rat.

#### Pro-inflammatory Genes and Receptors

In both species, TGFβ1 increased *Trem1* but decreased *Ccl3* and *Ccr5*. TGFβ1 decreased *Tnfa* and *Tnfrsf1b* only in rat and *Casp1* only in mouse. *Ifngr1* was oppositely affected by TGFβ1; increased in rat, decreased in mouse. IL-10 increased *Il1b* in mouse, while TGFβ1 decreased it in rat.

#### Additional Genes Related to Microglial Physiology

In both species, both cytokines increased *Fcgr2b*, and only TGFβ1 increased *Ncf1* and *Nox1*. In mouse, there were no other effects of TGFβ1; however, in rat, *P2rx7*, *Itgb2* and *P2ry12* were increased and *Fcgr3a* was decreased. In contrast, IL-10 increased *Fcgr3a* in both species. IL-10 also elevated *P2ry2* in rat, and *P2ry12* and *Msr1* in mouse.

#### Ion Channels and Regulators

TGFβ1 increased *Kcna3*, *Kcna5* and *Rest* in both species; *Kcnn4*, *Orai3* and *Trpm7* in rat; and *Kcnn3* in mouse. The only effect of IL-10 was reduced *Kcnj2* in mouse. For the K^+^ currents, there were some notable differences. In both species, the Kv1.3 current was unaffected by IL-10 (Lam et al., 2017) but increased by TGFβ1 in both neonatal and adult microglia (present study). Here, TGFβ1 did not affect the Kir2.1 (inward-rectifier) current in neonatal or adult microglia of either species; whereas, it was decreased by IL-10 in neonatal mouse microglia (Lam et al., 2017; KCa currents were not assessed in our recent study of IL-10).

## Discussion

We will summarize the present findings and relevant literature on rat and mouse under several topics. The emphasis will be on studies using primary rodent microglia because cell lines (e.g., BV-2 cells) differ in transcriptional responses (Butovsky et al., 2014; Das et al., 2016) and ion channel expression (Cayabyab et al., 2000; Ferreira and Schlichter, 2013; Liu et al., 2013).

### TGFβ1 in the Brain

TGFβ1 is a pleiotropic cytokine with immunosuppressive and anti-inflammatory properties (Colton, 2009; Cherry et al., 2014; Orihuela et al., 2016). TGFβ1 levels are often increased with brain damage or disease (see “Introduction” section), and it can act on microglia and other cells. We previously found that TGFβ1 expression is up-regulated in the damaged rat striatum in the first week after ICH or ischemia (Lively and Schlichter, 2012; Lively et al., 2016). It was also increased by intracerebral injection of IL-4 *in vivo* (Lively et al., 2016) or by adding IL-4 to rat microglia *in vitro* (Liu et al., 2013). A recent study of ICH in mice showed an increase in TGFβ1 *in vivo* (days 1–10), and an important role in reducing microglial inflammatory cytokine and chemokine expression *in vitro* (Taylor et al., 2017). In that study, microglia assumed a pro-inflammatory phenotype *in vivo* (expressing *Tnf*, *Il6*, *Ccl2*, *Cxcl10*) up to 3 days after ICH, and then during the recovery period (days 7–14), they up-regulated *Tgfb1*, *Tgfbr1* and *Tgfbr2*. Here, we found that 7 days after ICH in the rat, there was strong expression of the TGFβ1 receptor in microglia/activated macrophages within the hematoma.

### Effects of TGFβ1 on Microglia *in Vitro*

*In vitro*, microglia can be skewed to multiple anti-inflammatory phenotypes, which are usually assessed by molecular profiles and functional outcomes (reviewed in Colton, 2009; Cherry et al., 2014; Orihuela et al., 2016). Such states are increasingly defined by the stimulus (e.g., IL-4, IL-10, TGFβ1, glucocorticoids); however, it is not clear to what degree microglial responses to these stimuli differ and whether there are species differences.

#### Effects of TGFβ1 on Microglial Health

TGFβ1 is required for production of microglia in the mouse (Butovsky et al., 2014). Only a small number of *in vitro* studies have addressed whether TGFβ1 affects microglial health and survival. The results vary, but whether this reflects differences in protocols or species is not known. Stimulating primary mouse microglia with a wide range of TGFβ1 concentrations (0.001–1 ng/ml) did not affect proliferation 3 days later; but if proliferation was first stimulated by a mitogen (conditioned medium or granulocyte-macrophage colony-stimulating factor; GM-CSF), it was inhibited by as little as 0.01 ng/ml TGFβ1 (Suzumura et al., 1993). TGFβ1 inhibited proliferation when it was added to mouse microglia grown on a rat astrocyte bed (Jones et al., 1998). We found that 24 h treatment with 5, 10 or 20 ng/ml TGFβ1 did not affect primary mouse microglia proliferation (in agreement with Suzumura et al., 1993) but reduced proliferation of rat microglia by 20%–25%. We did not observe microglial apoptosis in either species at 24 h. In the only relevant study that we found of primary rat microglia, 10 ng/ml hTGFβ1 increased apoptosis at 48 h (Xiao et al., 1997); the 24 h time point was not mentioned.

#### TGFβ1-Induced Inflammatory Profile

Based mainly on mouse studies, it is thought that TGFβ1 can maintain a resting microglial state and can also contribute to alternative activation. IL-4-mediated up-regulation of *Ym1* and *Arg1* mRNA depended on TGFβ1 autocrine actions on Ras/MEK/ERK signaling (Zhou et al., 2012) and, conversely, inhibition of TGFβ1 signaling decreased Ym1 and Arg1 protein levels and up-regulated *Il6* and *iNOS* mRNA (Spittau et al., 2013). Broader transcriptional profiling of responses to TGFβ1 has recently begun for mouse microglia. An extensive microarray study applied TGFβ1 with CSF-1 (48 h), characterized the resulting state, which they called M0 because it was similar to freshly isolated adult microglia (Butovsky et al., 2014), and observed increases in 60 microglial genes, including *Tgfbr1*, *Tgfbr2*, *Kcnd1* (Kv4.1) and *P2ry12*. There were very few genes in common with the present study but qualitatively similar results following TGFβ1 treatment were seen for *Cx3cr1*, *Myc*, *Tgfbr1* and *Ccr5* in mouse microglia. Applying the blood product, thrombin, to cultured mouse microglia to model ICH, up-regulated *Il6*, *Tnf* and *Ccl2* and reduced *Tgfbr1*, and these changes were reversed by adding TGFβ1 (Taylor et al., 2017). Numerous *in vitro* studies have shown that TGFβ can interfere with pro-inflammatory signaling pathways induced by lipopolysaccharide (LPS; Kim et al., 2004; Le et al., 2004; Herrera-Molina and von Bernhardi, 2005; Naiki et al., 2005), and it inhibited LPS-, IFNγ- and GM-CSF-induced expression of major histocompatibility complex class II molecules (Loughlin et al., 1993; Suzumura et al., 1993; Lee et al., 1997; Hailer et al., 1998; O’Keefe et al., 1999). Such studies have led to TGFβ1 being proposed as a therapeutic strategy for treating CNS disease (reviewed in Cherry et al., 2014); however, few studies have compared microglial responses in rat and mouse (Patrizio and Levi, 1994; Du et al., 2017; Lam et al., 2017).

### Comparing Responses to TGFβ1 and IL-10

With CNS damage or disease, both TGFβ and IL-10 are released by multiple cell types, including microglia (De Simone et al., 2004; Minghetti et al., 2005) and astrocytes (Dobolyi et al., 2012; Villacampa et al., 2015). Historically, many researchers have implied that TGFβ and IL-10 evoke equivalent “acquired deactivation” of microglia and macrophages (reviewed in Colton and Wilcock, 2010; Cherry et al., 2014; Orihuela et al., 2016). However, very few studies have directly compared actions of the two cytokines on microglia. Two older studies examined their ability to resolve LPS-induced activation (Lodge and Sriram, 1996; Ledeboer et al., 2000). Co-treatment of mouse microglia with LPS and either TGFβ or IL-10 reduced TNFα and NO production, but only IL-10 inhibited LPS-induced IL-12 production (Lodge and Sriram, 1996). Simultaneous treatment of rat microglia/astrocyte co-cultures with LPS and a cocktail of TGFβ1 and TGFβ2 reduced NO production, but not release of IL-1β, TNFα or IL-6; whereas, IL-10 inhibited production of NO and pro-inflammatory cytokines (Ledeboer et al., 2000). Our transcription data on >50 genes comparing effects of TGFβ1 (present study) with IL-10 (Lam and Schlichter, 2015; Siddiqui et al., 2016; Lam et al., 2017) show important differences, despite some similarities. For instance, TGFβ1 reduced pro-inflammatory mediators more than IL-10 (e.g., *Casp1, Ccl3*, *Ccr5*, *Il1b*, *Ifngr1, Tnfa*, *Tnfrsf1b* were reduced in one or both species). TGFβ1 increased molecules involved in superoxide production (*Ncf1, Nox1*), while IL-10 increased several phagocytosis-related molecules (*Cd68*, *Mrc1, Fcgr3a, Msr1*). The two cytokines evoked opposite changes in *Cx3cr1*. Differing responses were also seen in channel activity. Notably, in both species, TGFβ1 increased Kv1.3 currents (present study) but IL-10 had no effect (Lam et al., 2017).

Differences in responsiveness to TGFβ1 and IL-10 were not accompanied by a deficiency or down-regulation of any of the cognate receptors in either species. Receptor transcript levels (*Il10ra*, *Il10rb*, *Tgfbr1*, *Tgfbr2*) were moderately high in unstimulated rat and mouse microglia, and not reduced by either of the cytokine treatments (Lam et al., 2017; present study). The finding that TGFβ1 treatment increased *Tgfbr1* and *Tgfbr2* expression suggests an increased capacity for autocrine anti-inflammatory signaling, which was not the case for IL-10 treatment. Instead, IL-10 increased expression of some pro-inflammatory receptors (*Tlr4*, *Tnfrsf1a*) as well as anti-inflammatory receptors (*Il4r*, *Il13ra1*, *Tgfbr2*), suggesting an overall increased ability to respond to subsequent inflammatory stimuli.

### Potassium Channel Expression and Function Is Affected By TGFβ1

Numerous microglial functions depend on Ca^2+^ signaling through CRAC (produced by Orai + Stim subunits) and SOC channels (Möller, 2002; Färber and Kettenmann, 2006), signaling that can be evoked by metabotropic purinergic (P_2_Y) receptors. K^+^ channels affect the membrane potential (Chung et al., 1998, 1999; Franchini et al., 2004; Newell and Schlichter, 2005) and regulate numerous microglia functions (Kettenmann et al., 2011; Stebbing et al., 2015). Here, TGFβ1 treatment did not affect the CRAC channel pore-forming subunit, *Orai1*, the CRAC regulator, *Stim1*, or* P2ry2* in either species. *Orai3* and *P2ry12* were up-regulated by TGFβ1 only in rat microglia. However, there were important effects on the K^+^ channels, each of which will be discussed separately with an emphasis on literature from primary rodent microglia. Even within studies on primary rodent microglia, there are considerable discrepancies in which K^+^ channel types are expressed, which currents are active, and what changes occur with different activation states. Recent patch-clamp studies of microglia in pro- and anti-inflammatory states have examined Kir2.1 in neonatal rat microglia (Lam and Schlichter, 2015), Kv1.3, Kir2.1 and KCa3.1 in neonatal mouse microglia (Nguyen et al., 2017a,b), and have compared Kv1.3 and Kir2.1 between neonatal mouse and rat microglia (Lam et al., 2017). From these and earlier studies, it is becoming clear that there are substantial qualitative and quantitative differences between the two species.

#### Kv1.3 Channels

An early hypothesis was that Kv current is induced by “activation” (Nörenberg et al., 1994; Fischer et al., 1995; Visentin et al., 1995; Illes et al., 1996) but the diversity of microglial states was not recognized nor were potential species differences. Kv currents can be up-regulated by pro-inflammatory stimuli (LPS, IFNγ, IFNγ+TNFα) in rat (Nörenberg et al., 1992, 1994; Visentin et al., 1995; Fordyce et al., 2005; Lam et al., 2017) and mouse microglia (Draheim et al., 1999; Prinz et al., 1999; Pannasch et al., 2006; Lam et al., 2017; Nguyen et al., 2017a). More than one Kv channel type has been detected in rodent microglia; e.g., Kv1.3 and Kv1.5 currents in *ex vivo* rat microglia (Kotecha and Schlichter, 1999) and LPS-stimulated mouse microglia (Pannasch et al., 2006). Thus, it is important to use selective blockers to identify Kv1.3. Using such blockers, we almost always detect robust Kv1.3 currents in unstimulated rat microglia (Schlichter et al., 1996; Cayabyab et al., 2000; Fordyce et al., 2005; Newell and Schlichter, 2005; Lam et al., 2017; present study). In unstimulated neonatal mouse microglia, we detected Kv1.3 currents less often, and they were small and accompanied by an additional Kv current (Lam et al., 2017; present study) that might be Kv1.5. In contrast, adult mouse microglia always had a Kv current, and it was predominantly Kv1.3, suggesting a developmental change. Of note, anti-inflammatory stimuli can also increase Kv1.3 expression and current in microglia; i.e., in response to IL-4 in rat (Lam et al., 2017), and TGFβ1 in neonatal mouse (Schilling et al., 2000) and neonatal and adult rat and mouse microglia (present study). Thus, Kv1.3 current is not specific to either a pro- or anti-inflammatory state and, instead, its up-regulation appears to signal a change from the resting, surveillance state. This might not be surprising, given that Kv1.3 is involved in numerous microglial functions that might occur in pro- or anti-inflammatory milieu, including proliferation (Kotecha and Schlichter, 1999), chemotactic migration (Nutile-McMenemy et al., 2007), chemokine and cytokine production (Liu et al., 2012; Charolidi et al., 2015), respiratory burst (Khanna et al., 2001), and even, neurotoxicity (Fordyce et al., 2005). The observed expression and roles need to be considered when targeting this channel *in vivo* to affect CNS inflammation.

#### Kir2.1 Channels

From the earliest recordings onward, a Kir2.1-like inward-rectifying current has usually been seen in unstimulated rat (Kettenmann et al., 1990; Korotzer and Cotman, 1992; Visentin et al., 1995; Schlichter et al., 1996) and mouse cells (Draheim et al., 1999; Prinz et al., 1999). But there are inconsistencies concerning its prevalence and amplitude in stimulated cells, and it is possible that they reflect heterogeneity in culture, stimuli or activation states. In response to LPS, IFNγ or IFNγ+TNFα, mouse microglia had smaller Kir currents (Draheim et al., 1999; Prinz et al., 1999; Boucsein et al., 2003; Lam et al., 2017) but in a recent study, LPS did not affect the current (Nguyen et al., 2017b). Rat cells varied from no change to a small increase with LPS, a large increase with IFNγ (Nörenberg et al., 1992, 1994; Visentin et al., 1995; Draheim et al., 1999), and a decrease with IFNγ+TNFα (Lam et al., 2017). The contrast with earlier studies using IFNγ might relate to our additional use of TNFα. While less is known about Kir2.1 responses to anti-inflammatory stimuli, the results also vary. In primary mouse microglia, IL-4-treated cells had large currents (Lam et al., 2017; Nguyen et al., 2017b), and the current was reduced by IL-10 (Lam et al., 2017) but not affected by TGFβ1 (Schilling et al., 2000; present study). In primary rat microglia, Kir2.1 was reduced by IL-4 (Lam et al., 2017) but unaffected by TGFβ1 in either neonatal or adult cells (present study). Roles of Kir2.1 are not well understood, possibly because the earlier blockers were not sufficiently potent or selective. In rat microglia, there is evidence that Kir2.1 regulates proliferation (Schlichter et al., 1996), and using a better blocker (ML133) we found that it regulates CRAC-mediated Ca^2+^ entry and ATP-induced chemotaxis (Lam and Schlichter, 2015). The observed decreases in Kir2.1 current by some pro- and anti-inflammatory stimuli, and possible species differences might make it difficult to target this channel for controlling microglial functions.

#### KCa3.1 Channels

KCa3.1 is considered a therapeutic target in the CNS because selective KCa3.1 blockers have improved the outcome in animal models of trauma, spinal cord injury, stroke and neurodegeneration (reviewed in Maezawa et al., 2012; Feske et al., 2015; Chen et al., 2016; Dale et al., 2016). Although *Kcnn4* transcripts have been reported in primary microglia both from rats (Khanna et al., 2001; Kaushal et al., 2007) and mice (Bouhy et al., 2011), the presence of a KCa3.1 current appears to be quite variable and dependent on the activation state. In rat microglia, small TRAM-34-sensitive KCa3.1 currents were recorded in a minority of unstimulated cells but transcripts and current were dramatically up-regulated by IL-4 (Ferreira et al., 2014) or TGFβ1 (present study). IL-4 up-regulated KCa3.1 through Ras/MEK/ERK signaling, and the channel then contributed to migration, invasion and Ca^2+^ signaling (Ferreira et al., 2014). However, KCa3.1 also regulates LPS-mediated activation (through p38 MAPK), leading to production of pro-inflammatory mediators and neuron killing (Kaushal et al., 2007; Liu et al., 2013), and UTP-stimulated chemotaxis and expression of the pro-inflammatory genes, IL-1β and iNOS (Ferreira and Schlichter, 2013). In mouse microglia, KCa3.1 currents are also variable, and apparently susceptible to culturing conditions and activation states but effects of TGFβ1 were not studied. In early studies, moderate charybdotoxin-sensitive KCa currents were seen when microglia from NMRI mice were cultured with M-CSF and astrocyte-conditioned medium (Eder et al., 1997; Schilling et al., 2002). Later studies using the more selective blocker, TRAM-34, showed that unstimulated cells had small (Maezawa et al., 2011; Nguyen et al., 2017b) to moderate KCa3.1 currents (present study). Intriguingly, the current was unaffected by IL-4 or LPS alone, and moderately increased by IFNγ but not by IFNγ + LPS (Nguyen et al., 2017b), while we found that it was greatly decreased by TGFβ1 (present study). Variable KCa3.1 expression was seen in *ex vivo* tissue from damage models. For instance, no KCa3.1 current was seen in juvenile mouse microglia within acute brain slices (Schilling and Eder, 2007); whereas in adult microglia isolated using CD11b-coated magnetic beads, there were KCa3.1 currents in some microglia at 8 days after ischemia, and in most microglia isolated from LPS-injected mouse brains (Chen et al., 2016). In adult human microglia from epilepsy patients, most cells expressed a KCa3.1 current, which was unaffected by LPS and slightly increased by IL-4 (Blomster et al., 2016). Thus, a clear picture of KCa3.1 expression in microglia has not emerged.

## Conclusions and Future Directions

Studies of disease mechanisms rely heavily on rodent models and translation to humans is a continuing challenge. Transcriptional profiling is increasingly used to investigate microglia in CNS damage and disease states, and an important question is whether differences reported in microglia responses *in vivo* and *in vitro* instead reflect species differences. Our *in vitro* study of >50 targeted molecules showed significant species similarities as well as important differences in microglial responses to TGFβ1. Some genes can be compared with recent high-content studies analyzing microglia isolated from both naive and damaged or diseased brains. Most of those studies were on mice, with some exemplary molecules validated in human brain tissue. Molecular signatures include homeostatic genes expressed in normal microglia (Gautier et al., 2012; Orre et al., 2014; Zhang et al., 2014), a microglial “sensome” in adult and aged mice (Hickman et al., 2013), and a unique plaque-associated phagocytic subtype in Alzheimer’s (“Disease-associated microglia”/DAM; Keren-Shaul et al., 2017; Krasemann et al., 2017). Several molecules that we found were well expressed in control microglia and up-regulated by TGFβ1 are in a homeostatic fingerprint that is maintained by TGFβ1 signaling (Butovsky et al., 2014; Gosselin et al., 2014); e.g., *Csf1r*, *Cx3cr1*, *P2ry12*. Several microglial signature molecules were also restricted to microglia in humans; i.e., *C1qa*, *Gas6*, *Mertk*, *P2ry12*, and *Pros1* (Butovsky et al., 2014). Some molecules we examined (including *P2ry12*, C*sf1r*, *Cx3cr1*, *Itgam* (CD11b), *Cd68*, *Tlr2*, *Itgb2*, and various Fc receptors) are part of the “sensome”, which is a large group of molecules and receptors at the cell surface involved in microglia sensing environmental perturbations, as in damage or disease (Hickman et al., 2013). Some sensome-related molecules that were increased by TGFβ1 in our study are commonly down-regulated in AD-associated microglia and DAM (Keren-Shaul et al., 2017; Krasemann et al., 2017); e.g., *Csf1r*, *Cx3cr1*, *P2ry12*, *Tgfbr1*.

Goals of molecular fingerprinting studies include: selecting and interpreting markers and molecules in animal models of CNS disease; identifying a reliable panel of human markers to help define disease progress and determine whether treatments are effective; and identifying molecular targets to control neuroinflammation. To this end, it is essential to compare human microglia with rodent models; however, there is very little published data comparing human responses to TGFβ1. A recent article raises concerns because a hallmark TGFβ1 response in rodents was not seen in human microglia (Smith et al., 2013). The authors investigated human leukocyte antigen (HLA) molecules, which are human analogs of the MHC II complex. IFNγ increases HLA expression and TGFβ1 blocks this response in rodent microglia; however, in adult human microglia, TGFβ1 (10 ng/ml) was completely ineffective. In future, it will be crucial to compare a wide range of TGFβ1 responses between human and rodent microglia.

## Data Availability

The raw data supporting the conclusions of this manuscript will be made available by the authors, without undue reservation, to any qualified researcher.

## Author Contributions

The project was mainly conceived and spearheaded by LCS, with important contributions from SL, DL and RW. SL carried out the analyses of ICH tissue, Nanostring, Western blotting and functional studies (morphology, proliferation, apoptosis; Tables 1–4, Figures 1, 2, 8, Supplementary Tables S3, S4). DL and RW conducted all the patch-clamp electrophysiology and analysis (Figures 3–7). LCS and SL wrote the manuscript, with input from DL and RW.

## Conflict of Interest Statement

The authors declare that the research was conducted in the absence of any commercial or financial relationships that could be construed as a potential conflict of interest. The reviewer RR and handling Editor declared their shared affiliation.

## Footnotes

^1^https://imagej.nih.gov/ij/

^2^https://www.pmgenomics.ca/pmgenomics
