# Supplementary material for: Comparing Effects of Transforming Growth Factor β1 on Microglia From Rat and Mouse: Transcriptional Profiles and Potassium Channels
Source: Front Cell Neurosci. 2018 May 3;12:115. doi: 10.3389/fncel.2018.00115 (PMC5946019; doi:10.3389/fncel.2018.00115)
Supplement: Supplementary file 1 [file Table_1.PDF]

Comparing effects of transforming growth factor b1 on microglia from rat and mouse: Transcriptional profiles and potassium channels

Starlee Lively, Doris Lam, Raymond Wong and Lyanne C. Schlichter*
* Correspondence: Professor Lyanne C. Schlichter Lyanne.Schlichter@uhnresearch.ca

Supplementary Table 1. Rat target sequences for nCounter Assay CodeSet

Gene	Accession #	Target sequence
Adora1	NM_017155.2	TGTCCTCATCCTCACCCAGAGCTCCATTCTGGCTCTGCTCGCCATTGCTG TGGATCGATACCTCCGAGTCAAGATCCCTCTCCGGTACAAGACAGTGGTG
Adora2a	NM_053294.3	CCCTGGAGAAGTCATGATTTTGGGAGATGCAGAGTCGTCTGTGAGAACGT CTTCGGGGAGCTCTCTCTGGGAACGCTCGTGGCTCTTGTGAGGAAAGGGC
Aif1 (Iba1)	NM_017196.2	ATCGATATTATGTCCTTGAAGCGAATGCTGGAGAAACTTGGGGTTCCCAA GACCCATCTAGAGCTGAAGAAATTAATTAGAGAGGTGTCCAGTGGCTCCG
Arg1	NM_017134.2	ACGGGAAGGTAATCATAAGCCAGAGACTGACTACCTTAAACCACCGAAAT AAATGTGAATACATCGCATAAAAGTCATCTGGGGCATCACAGCAAACCGA
Calm	NM_031969.2	GCTACATCAGTGCGGCAGAACTGCGCCACGTCATGACAAACCTCGGGGAA AAGCTAACAGATGAAGAAGTAGACGAAATGATCAGAGAAGCAGATATTGA
Casp1 (ICE)	NM_012762.2	AGATTCTAAGGGAGGACATCCTTTCTCCTCAGAAACAAAAGAAAAACTGA ACAAAGAAGGTGGCGCATTTCCTGGACCGAGTGGTTCCCTCAAGTTTTGC
Ccl3	NM_013025.2	ACGGCAAATTCCACGAAAATTCATTGCTGACTATTTTGAGACCAGCAGCC TTTGCTCCCAGCCGGGTGTCATTTTCCTGACCAAGAGAAACCGGCAGATC
Ccl22	NM_057203.1	TACATCCGTCACCCTCTGCCACCACGTTTCGTGAAGGAGTTCTACTGGAC CTCAAAGTCCTGCCGCAAGCCTGGCGTCGTTTTGATAACCATCAAGAACC
Ccr2	NM_021866.1	ATTCTCTATTTCCAAGAAGTATCCAAGAGCTTGATGAGGGGGCCACCACA CCGTATGACTATGATGATGGTGAACCTTGTCATAAAACCAGTGTGAAGCA
Ccr5	NM_053960.3	AGGACATGAAGAGAATGGTCAAGGCCGAATCCTAAGGCATCATATCAGCA TTGCTTCTTCAGAGTTATGAGCATGGTGGGGAGCACCTAGACAGGTTTGT
Cd68 (ED1)	NM_001031638.1	CTCTCATTCCCTTACGGACAGCTTACCTTTGGATTCAAACAGGACCGACA TCAGAGCCACAGTACAGTCTACCTTAACTACATGGCAGTGGAATACAATG
Cd163	NM_001107887.1	CCTCTGTAATTTGCTCAGGAAACCAATCGCATACACTGTTGCCATGTAGT TCATCATCTTCGGTCCAAACAACAAGTTCTACCATTGCAAAGGACAGTGA
Csf1r	NM_001029901.1	ACCTACCTAGGGCCGTTCTTTGAAGACCCACACAATCTTGAGTTTAGAAC CCAATGGACCACATACAGCTACTCATTCAAACTCCACCTGAACCGTGTAA
Cx3cr1	NM_133534.1	ATGTGCAAGCTCACGACTGCTTTCTTCTTCATTGGCTTCTTTGGGGGCAT ATTCTTCATCACCGTCATCAGCATCGACCGGTACCTCGCCATCGTCCTGG
Cybb (Nox2)	NM_023965.1	CAGTACCAAAGTTTGCCGGAAACCCTCCTATGACTTGGAAATGGATCGTG GGTCCCATGTTCCTGTATCTGTGTGAGAGGCTGGTGCGGTTTTGGCGATC
Fcgr1a	NM_001100836.1	TGATGGATCATACTGGTGCGAGGTAGCCACGGAGGACGGCCGTGTCCTTA AGCGCAGCACCAAGTTGGAGCTATTTGGTCCCCAGTCATCAGATCCTGTC
Fcgr2b	NM_175756.1	CTGGTCCAAGGAATGCTGTAGATATGAAAGAAAACATCTAGAGTCCCTTC TGTGAGTCCTGAAACCAACAGACACTACGATATTGGTTCCCAATGGTTGA
Fcgr3a	NM_207603.1	GACTCTTGTTTGCAATAGACACAGTGCTGTATTTCTCGGTGCAGAGGAGT CTTCAAAGTTCCGTGGCAGTCTATGAGGAACCCAAACTTCACTGGAGCAA
Gusb	NM_017015.2	TCATTTGATCCTGGATGAGAAACGAAAAGAATATGTCATCGGAGAGCTCA

		TCTGGAATTTTGCTGACTTCATGACGAACCAGTCACCACTGAGAGTAACA
Hprt1	NM_012583.2	AGCTTCCTCCTCAGACCGCTTTTCCCGCGAGCCGACCGGTTCTGTCATGT CGACCCTCAGTCCCAGCGTCGTGATTAGTGATGATGAACCAGGTTATGAC
Hvcn1	XM_006249369.2	ACCAAGAGGATGAGCAGGTTCTTGAAGCACTTCACAGTGGTGGGGGACGA CTACCACACCTGGAATGTCAACTACAAGAAGTGGGAGAACGAGGAGGATG
Ifng	NM_138880.2	AAGGACGGTAACACGAAAATACTTGAGAGCCAGATTATCTCTTTCTACCT CAGACTCTTTGAAGTCTTGAAAGACAACCAGGCCATCAGCAACAACATAA
Ifngr1	NM_053783.1	CCTGTTACACATTCGACTACACTGTGTTTGTGAAACATTACAGGAGTGGG GAGATCCTACATACAGAACATAGCGTCCTAAAAGAAGATTGTAGCGAAAC
Ifngr2	NM_001108313.1	TTTCTTAAGTTACACTTAGTAAAGCAGATGAGTCCGCAGGAGACTTCAGC AAGAAAGAAGTTCCTACCGTCTCATCCCTTAGTTCTTCAAAGCCAAAGGA
Il1b	NM_031512.1	TGCACTGCAGGCTTCGAGATGAACAACAAAAATGCCTCGTGCTGTCTGAC CCATGTGAGCTGAAAGCTCTCCACCTCAATGGACAGAACATAAGCCAACA
Il1r1	NM_013123.3	CTCATATTCTGGAGACTGCACACGTACGGTTAGTATACCCAGTTCCTGAC TTCAAGAATTACCTCATCGGGGGCTTTGCCATCTTCACAGCTACAGCCGT
Il1rn	NM_022194.2	TCATTGCTGGGTACTTACAAGGACCAAATACCAAACTAGAAGAAAAGATA GACATGGTGCCTATTGACTTTCGGAATGTGTTCTTGGGCATCCACGGGGG
Il4	NM_201270.1	TGCTGTCACCCTGTTCTGCTTTCTCATATGTACCGGGAACGGTATCCACG GATGTAACGACAGCCCTCTGAGAGAGATCATCAACACTTTGAACCAGGTC
Il4r	NM_133380.2	GGGTGTCAGCATCTCCTGCATCTGCATCCTATTGTTTTGCCTGACCTGTT ACTTCAGCATTATCAAGATTAAGAAGATATGGTGGGACCAGATTCCCACT
Il6	NM_012589.1	GGAACAGCTATGAAGTTTCTCTCCGCAAGAGACTTCCAGCCAGTTGCCTT CTTGGGACTGATGTTGTTGACAGCCACTGCCTTCCCTACTTCACAAGTCC
Il10	NM_012854.2	ACAACATACTGCTGACAGATTCCTTACTGCAGGACTTTAAGGGTTACTTG GGTTGCCAAGCCTTGTCAGAAATGATCAAGTTTTACCTGGTAGAAGTGAT
Il10ra	NM_057193.2	TGTTTACATGTCACGACGGAGCATTATTTCACCGTGACCAACCTCAGCAT TTTCTTCTTATCCATCCTGATACTCTGTGGAGCCCTGGTCTGCCTGGTTC
Il10rb	NM_001107111.1	CCTCCCTGGATCGTGGCCATCATCCTTATAGCCTCCGTCTTGATAGTCTT CCTCTTCCTACTGGGCTGCTTCAGCATGGTGTGGTTCATTTACAAGAAGA
Il13ra1	NM_145789.2	TAACGAATTTGAGTGTCTCTGTCGAAAATCTCTGCACAATAGTGTGGACA TGGAGTCCTCCTGAGGGAGCCAGTCCAAATTGCAGTCTCAGATATTTTAG
Itgam (Cd11b)	NM_012711.1	CATCCCTTCCTTCAACAGTAAAGAAATATTCAACGTCACCCTCCAGGGCA ATCTGCTATTTGACTGGTACATCGAGACTTCTCATGACCACCTCCTGCTT
Itgb2	NM_001037780.2	CAATATCAGGTGCAACGGAGTCAACTGTCTCCACAAAAAGTGACCCTTAA CTTGCGACCAGGGCAGGCTGCTGCATTCAATGTGACTTTCCGACGGGCCA
Kcna2 (Kv1.2)	NM_012970.3	GCCGGCCAGGATCATAGCCATTGTATCTGTGATGGTCATTCTGATCTCGA TCGTCAGCTTCTGTCTGGAAACCTTGCCCATCTTCCGGGATGAGAACGAG
Kcna3 (Kv1.3)	NM_019270.3	GCCACCTTCTCCAGAAATATCATGAACCTGATAGACATTGTAGCCATCAT CCCTTATTTTATTACTCTGGGCACTGAGCTGGCTGAGCGACAGGGTAATG
Kcna5 (Kv1.5)	NM_012972.1	ATCAGAAGGGGTAGCTGTCCTCTAGAAAAGTGTCACCTCAAGGCCAAGAG CAACGTGGACTTGCGGAGGTCCCTGTATGCCCTCTGTCTGGACACTAGCC
Kcnj2 (Kir2.1)	NM_017296.1	GTTCTTTGGCTGTGTGTTTTGGTTGATAGCTCTGCTCCACGGGGATCTGG ATGCTTCTAAAGAGAGCAAAGCGTGTGTGTCTGAGGTCAACAGCTTCACG
Kcnma1 (Bk)	NM_031828.1	TGATATCTGCCCAGACACTGACTGGCAGAGTCCTGGTTGTGTTAGTCTTT GCTCTCAGCATTGGTGCCCTTGTAATATACTTCATAGACTCATCAAACCC
Kcnn3 (SK3)	NM_019315.2	AGAGAAAGCGACTGAGTGACTATGCTCTGATTTTTGGGATGTTTGGAATT GTTGTTATGGTGATAGAGACCGAACTGTCTTGGGGTTTGTACTCAAAGGA

Kcnn4 (SK4)	NM_023021.2	TACGTCTCTACCTGGTGCCTCGCGCGGTACTTCTGCGTAGCGGGGTCCTG<br>CTCAACGCGTCTTACCGCAGCATCGGGGCGCTCAACCAAGTCCGATTCCG
Mrc1 (CD206)	NM_001106123.1	CTTTGGAATCAAGGGCACAGAGCTATATTTTAACTATGGCAACAGGCAAG<br>AAAAGAATATCAAGCTTTACAAAGGTTCCGGTTTGTGGAGCAGATGGAAG
Msr1 (SR-A)	NM_001191939.1	CACGTTCCATGACAGCATCCCTTCCTCACAACACTATAAATGGCTCCTCC<br>GTTCAGGAGAAACTGAAGTCCTTCAAAGTTGCCCTCGTCGCTCTCTACCT
Myc	NM_012603.2	ACCGAGGAAAACGACAAGAGGCGGACACACAACGTCTTGGAACGTCAGAG<br>GAGAAACGAGCTGAAGCGTAGCTTTTTTGCCCTGCGCGACCAGATCCCTG
Ncf1	NM_053734.2	TCCATTCCCAGCATCCCATAATTGGGCTTGTCCGTGTTCCAACATCTGGG<br>CGGAATTTCACAGCCAAAGGTCAAGAGGACTGCTGTTACGTTCAAGGTCG
Nfkbia (IκBα)	NM_001105720.2	TATTGTGCTTTTGGTTGAACCGCCATAGACTGTAGCTGACCCCAGTGTGC<br>CCTCTCACGTAAGAACCAGGTGTTCAGTGGTATGTGCTTAAGTCATCCCC
Nme2 (NDPK-B)	NM_031833.2	TGATTCAGTGGAGAGTGCCGAGAAAGAGATCGGTCTATGGTTTAAGCCCG<br>AAGAACTGATTGACTATAAGTCTTGTGCCCATGACTGGGTGTATGAGTAG
Nos2	NM_012611.2	ACGGGACACAGTGTCGCTGGTTTGAAACTTCTCAGCCACCTTGGTGAGGG<br>GACTGGACTTTTAGAGACGCTTCTGAGGTTCCTCAGGCTTGGGTCTTGTT
Nox1	NM_053683.1	CCGAGAAAGAAGATTCTTGGCTAAATCCCATCCAGTCTCCAAACGTGACA<br>GTGATGTATGCAGCATTTACCAGTATTGCTGGCCTTACTGGAGTGGTCGC
Nox4	NM_053524.1	TGTTGGACAAAAGCAAGACTCTACATATCACCTGTGGCATAACTATTTGT<br>ATTTTCTCAGGTGTGCATGTAGCTGCCCACTTGGTGAACGCCCTGAACTT
Nr3c1 (GR)	NM_012576.2	AGCTTTCCTTGAAGCGTATAAAGAGCCATGCTCCTTTAGTATGTGGGGAA<br>GAAGAGAGCTGTCATAGTTTTGAGTACAGTGAGAAGATGCGGTACTGTCT
Orai1	NM_001013982.1	GCCTTCTCCACCGTCATCGGGACGCTGCTTTTCCTGGCCGAAGTCGTGCT<br>GCTCTGCTGGGTGAAGTTCTTACCGCTCAAGAGGCAGGCGGGACAGCCAA
Orai3	NM_001014024.1	ACCTGTAATGTGCTTTACAGTTGGCATCCTGGGAGAGATTTTACATAGGC<br>TCCTCAGATGAACCACTTTACACTTGGTGACTTGTGGTGGTGTGTCCCAC
P2rx7	NM_019256.1	ACTTTAAGAGGTCACATTAACCAGACTAGAAGCCATCGCATCTAACCGCA<br>TACCAGACACAGTCTGACGCCTCATTGCTATGCTATGGTTCTAAGTGACT
P2ry2	NM_017255.1	GAGCTCTTTAGCCATTTTGTGGCTTACAGCTCTGTCATGCTGGGTCTGCT<br>TTTTGCTGTGCCCTTTTCCATCATCCTGGTCTGTTACGTGCTCATGGCCC
P2ry12	NM_022800.1	TGATAACCATTGACCGATACCTGAAGACCACCAGACCATTTAAAACTTCC<br>AGCCCCAGCAATCTTTTGGGTGCGAAGATTCTTTCTGTTGCCATCTGGGC
Phtp1	NM_001106558.2	CCTGTGTTCTCCTTTTGTACCTTGGGCAACACACCACCTGCCAGGCCTTA<br>GAGGCCAGAGCAATCTGATCCATAGGAATTAAAGTATTGATATGCCTACT
Pparg	NM_013124.1	TTTATAGCTGTCATTATTCTCAGTGGAGACCGCCCAGGCTTGCTGAACGT<br>GAAGCCCATCGAGGACATCCAAGACAACCTGCTGCAGGCCCTGGAACTCC
Ptgs2 (Cox2)	NM_017232.3	TTCGGAGGAGAAGTGGGTTTTAGGATCATCAACACTGCCTCAATTCAGTC<br>TCTCATCTGCAATAATGTGAAAGGGTGTCCCTTTGCCTCTTTCAATGTGC
Ptk2b (Pyk2)	NM_017318.2	GCAGTGATCATGAAGAATCTTGACCACCCTCACATCGTCAAGCTGATTGG<br>CATCATTGAAGAGGAACCCACATGGATCGTCATGGAACTGTATCCTTATG
Ptpn6 (SHP-1)	NM_053908.1	GCAGAGTCACTGCTGCAGGCCAAGGGCGAGCCCTGGACATTTCTTGTGCG<br>TGAGAGTCTCAGCCAACCTGGTGATTTTGTGCTCTCTGTGCTCAATGACC
Rest	NM_031788.1	GCTGAGCTGGCTGCTCCCATGGAATCTACCAGTGCTTTATCCTCTGAACA<br>AAGCTCAAATGCACCAGATGGTGAAACATTACACAGCGAGTGTCAGGCTG
Retnla (Fizz1)	NM_053333.1	AGGAACTTCTAGCCCATCAAGATAACTATCCCTCTGCTGTAAGGAAGACC<br>CTCTCATGCACTAATGTCAAGTCTATGAGCAAATGGGCCTCCTGCCCTGC
Socs1	NM_145879.1	CGGCCGCTGCAGGAGCTGTGTCGCCAGCGCATCGTGGCCGCCGTGGGTCG

		CGAGAACCTGGCACGCATCCCTCTTAACCCGGTACTCCGTGACTACCTGA
Socs3	NM_053565.1	GGAAGACTGTCAACGGTCACCTGGACTCCTATGAGAAAGTGACCCAGCTG<br>CCTGGACCCATTCGGGAGTTCCTGGACCAGTATGATGCTCCACTTTAAAG
Stim1	NM_001108496.2	TATCTATCGTGATTGGTGTGGGTGGCTGCTGGTTTGCCTATATCCAGAAC<br>CGTTACTCTAAGGAGCACATGAAGAAAATGATGAAGGATCTGGAAGGATT
Tgfb1	NM_021578.2	CGCCTGCAGAGATTCAAGTCAACTGTGGAGCAACACGTAGAACTCTACCA<br>GAAATATAGCAACAATTCCTGGCGTTACCTTGGTAACCGGCTGCTGACCC
Tgfbr1	NM_012775.2	GTCTGCATTGCACTTATGCTGATGGTCTATATCTGCCATAACCGCACTGT<br>CATTCACCACCGCGTACCAAATGAAGAGGATCCCTCACTAGATCGCCCTT
Tgfbr2	NM_031132.3	CCAGCAGTCCTGACCTGTTGCTGGTCATTATCCAAGTGACGGGCGTCAGC<br>CTCCTGCCTCCGCTGGGGATTGCCATAGCTGTCATTGCCATCTTCTACTG
Tlr2	NM_198769.2	TTTACAAACCCTTAGGGTAGGAAATGTTGACACTTTCAGTGAGATAAGGA<br>GAATAGATTTTGCTGGGCTGACCTCTCTCAACGAACTTGAAATTCAGGTA
Tlr4	NM_019178.1	GTCAGTGTGCTTGTGGTAGCCACTGTAGCATTTCTGATATACCACTTCTA<br>TTTTCACCTGATACTTATTGCTGGCTGTAAAAAGTACAGCAGAGGAGAAA
Tnf	NM_012675.2	GGTGATCGGTCCCAACAAGGAGGAGAAGTTCCCAAATGGGCTCCCTCTCA<br>TCAGTTCCATGGCCCAGACCCTCACACTCAGATCATCTTCTCAAAACTCG
Tnfrsf1a (TNFR1)	NM_013091.1	TATTCTTTATCTGCATCAGTCTACTGTGCCGATATCCCCAGTGGAGGCCC<br>AGGGTCTACTCCATCATTTGTAGGGATTCAGCTCCTGTCAAAGAGGTGGA
Tnfrsf1b (TNFR2)	NM_130426.4	AGGAGTTCAGATTCTTCCCATGGCAGCCACGGGACCCATGTCAACGTCAC<br>CTGCATCGTGAACGTCTGTAGCAGCTCTGACCACAGCTCTCAGTGTTCTT
Trem1	NM_001106885.1	TCAAATGACTGACCTTCAAGTGACAGACTCTGGATTATATCGTTGTGTGA<br>TTTACCATCCTCCGAACGACCCTGTTCTGCTCTTCCATCCCGTCCGCCTG
Trem2	NM_001106884.1	TCCGGCTGGCTGAGGAAGGGTGCCATGGAACCTCTCCACGTGTTTGTCCT<br>GTTGCTGGTCACAGAGCTGTCCCAAGCCCTCAACACCACAGTGCTGCAGG
Trpm2	NM_001011559.1	GTGAGCCTCCGGTCTCTCTATAAGCGATCAACAGGCCACGTTACCTTCAC<br>CATTGACCCAGTCCGCGATCTTCTCATTTGGGCCATCATCCAGAACCACA
Trpm4	NM_001136229.1	AACTATTCTGCTTTCTTCTTGGTGGATGATGGTACCTATGGCCGCATGGG<br>TGGTGAGAACCGCTTCCGCCTTCGGTTTGAGTCCTATGTGGCTCAGCAGA
Trpm7	XM_001056331.1	TTCTCTTCATTCAGAAACAGAGAGCTGTAGTAGAAGAGCGTCGACAGAAG<br>ACTCTCCGGACGTAGATTCCAGAGCAGCTTTGTTGCCGGATTGGTTACGA
Tspo	NM_012515.1	GCTGCCCGCTTGCTGTATCCTTACCTGGCCTGGCTGGCCTTTGCCACCAT<br>GCTCAACTACTATGTATGGCGTGATAACTCTGGTCGGCGAGGGGGCTCCC
