# Supplementary material for: Comparing Effects of Transforming Growth Factor β1 on Microglia From Rat and Mouse: Transcriptional Profiles and Potassium Channels
Source: Front Cell Neurosci. 2018 May 3;12:115. doi: 10.3389/fncel.2018.00115 (PMC5946019; doi:10.3389/fncel.2018.00115)
Supplement: Supplementary file 2 [file Table_2.PDF]

Comparing effects of transforming growth factor b1 on microglia from rat and mouse: Transcriptional profiles and potassium channels

Starlee Lively, Doris Lam, Raymond Wong and Lyanne C. Schlichter*
* Correspondence: Professor Lyanne C. Schlichter Lyanne.Schlichter@uhnresearch.ca

Supplementary Table 2. Mouse target sequences for nCounter Assay CodeSet

Gene	Accession #	Target sequence
Adora1	NM_001008533.3	TGCCTCTGAGACCTGAGTGGCTTTCCTTTTTAAACACAAAATGCTCACGG<br>CTGTGACACTCTGGCTGATGTTAAAGAGTATGGCTTCTAGGGTGAGTGAG
Adora2a	NM_009630.2	ACTATGAAAGGCAGGGTGCCAGAGTGTGGGCTCACGTCTCAGGATTGAGT<br>TTAGAGACATCAAGTCATGGACCCGAGCTGGATAGTTCAGAGCTGCCCTG
Aif1 (Iba1)	NM_019467.2	CTGGAGCAGCCTGCAGACTTCATCCTCTCTCTTCCATCCCGGGGAAAGTC<br>AGCCAGTCCTCCTCAGCTGCCTGTCTTAACCTGCATCATGAAGCCTGAGG
Arg1	NM_007482.3	GTACATTGGCTTGCGAGACGTAGACCCTGGGGAACACTATATAATAAAAA<br>CTCTGGGAATTAAGTATTTCTCCATGACTGAAGTAGACAAGCTGGGGATT
Calm	NM_009790.4	GGTCAGCATACACAGAGACACGCAACATCCAGTGCAAGCTGGATTTCCCA<br>CCAGGTTCTCTAGCCACAACTCCTGAAACCAGACAGACTCGGAGTTCTTT
Casp1 (ICE)	NM_009807.2	GACAATAAATGGATTGTTGGATGAACTTTTAGAGAAGAGAGTGCTGAATC<br>AGGAAGAAATGGATAAAATAAAACTTGCAAACATTACTGCTATGGACAAG
Ccl3	NM_011337.1	TCTGTCACCTGCTCAACATCATGAAGGTCTCCACCACTGCCCTTGCTGTT<br>CTTCTCTGTACCATGACACTCTGCAACCAAGTCTTCTCAGCGCCATATGG
Ccl22	NM_009137.2	CCAAGAATCAACTTCCACCCCTCTTCAACCACATGCTAGGGTCTTTTACT<br>TTCTCTGCCCCACACCTTTGACTCCTTGCCTGTGTAGCTGATAGTCGAAG
Ccr2	NM_009915.2	ATGAACTAACATAGACAGCTCAGGATTAACAGGGACTTGTGGTTTGTGGT<br>CTGTGGGCTTATCCAAGCATGGTGATTTAGACTCTAAGGTCCGTCTGGAT
Ccr5	NM_009917.5	GGAGCAGGGAGAACGAGTCTTTTATCAGGGCCGGGAAATATGCACAAAGA<br>GACTTGAGGCAGGTGCCATGACCCATATGCAAAGGGACGGACACAGGGCC
Cd68 (ED1)	NM_009853.1	GCTCCCTGTGTGTCTGATCTTGCTAGGACCGCTTATAGCCCAAGGAACAG<br>AGGAAGACTGTCCTCACAAAAAGGCCGTTACTCTCCTGCCATCCTTCACG
Cd163	NM_053094.2	TCACGGCACTCTTGGTTTGTGGAGCCATTCTATTGGTCCTCCTCATTGTC<br>TTCCTCCTGTGGACTCTGAAGCGACGACAGATTCAGCGACTTACAGTTTC
Csf1r	NM_001037859.2	GTACAAGCAGAAGCCGAAGTACCAGGTGCGCTGGAAGATCATCGAGAGAT<br>ACGAAGGCAATAGCTACACCTTCATTGACCCTACTCAGTTGCCCTACAAT
Cx3cr1	NM_009987.3	TATGCTTTGGTGTTGGTCTGTATTTCCCGCTGTCTCGGGTCACATGGTTA<br>AGCGTGCCTAGAGTGTGTCTATCCCACTTGTAATTCTGTCAATAAACATT
Cybb (Nox2)	NM_007807.2	ACAGAAGACTCTGTATGGACGGCCCAACTGGGATAACGAGTTCAAGACCA<br>TTGCAAGTGAACACCCTAACACCACAATAGGCGTTTTCCTGTGTGGCCCT
Fcgr1a	NM_010186.5	GAGACAGTTCCACACAATGGTTTATCAACGGAACAGCCGTTCAGATCTCC<br>ACGCCTAGTTATAGCATCCCAGAGGCCAGTTTTCAGGACAGTGGCGAATA
Fcgr2b	NM_001077189.1	TTGGTTCCCAATGGTTGACTGTACTAATGACTCCCATAACTTACAGCTTC<br>CCAACTCAAGACTCTTCTGCTATCGATCCACACTGCCACTAAAATTAATC
Fcgr3a	NM_010188.5	TCTGACCTCCACCATCCACCATGGCAGGTGCACACAATAAATTAAAATGT<br>CATGTATATTTTTAAACAAGAGACAGGGGCAGGCTAAGGGTTGATGGCAT
Gusb	NM_010368.1	AATACGTGGTCGGAGAGCTCATCTGGAATTTCGCCGACTTCATGACGAAC<br>CAGTCACCGCTGAGAGTAATCGGAAACAAGAAGGGGATCTTCACTCGCCA

Hprt1	NM_013556.2	TGCTGAGGCGGCGAGGGAGAGCGTTGGGCTTACCTCACTGCTTTCCGGAG CGGTAGCACCTCCTCCGCCGGCTTCCTCCTCAGACCGCTTTTTGCCGCGA
Hvcn1	NM_001042489.1	GTACCATCAGCTTCAAACTGGCTCTGTACCCACTAGCAGCTCTTTCAGTT ACTGTACAATTAATGTGTAAAATAAGCCCTCTCCTTCCCAGGGCTCACCC
Ifng	NM_008337.1	CTAGCTCTGAGACAATGAACGCTACACACTGCATCTTGGCTTTGCAGCTC TTCCTCATGGCTGTTTCTGGCTGTTACTGCCACGGCACAGTCATTGAAAG
Ifngr1	NM_010511.2	AAGCATAATGTTACCTAAGTCCTTGCTCTCTGTGGTAAAAAGTGCCACGT TAGAGACAAAACCTGAATCGAAGTATTCACTTGTCACACCGCACCAGCCA
Ifngr2	NM_008338.3	CATCCTGATTCCGTTGGGCATCTTCGCATTGCTGCTCGGCCTGACGGGCG CCTGCTTCACCCTGTTCCTCAAATACCAAAGCCGAGTGAAGTACTGGTTT
Il1b	NM_008361.3	GTTGATTCAAGGGGACATTAGGCAGCACTCTCTAGAACAGAACCTAGCTG TCAACGTGTGGGGGATGAATTGGTCATAGCCCGCACTGAGGTCTTTCATT
Il1r1	NM_001123382.1	CTTCTTCGGAGTAAAAGATAAACTGTTGGTGAGGAATGTGGCTGAAGAGC ACAGAGGGGACTATATATGCCGTATGTCCTATACGTTCCGGGGGAAGCAA
Il1rn	NM_031167.5	CAACCAGCTCATTGCTGGGTACTTACAAGGACCAAATATCAAACTAGAAG AAAAGATAGACATGGTGCCTATTGACCTTCATAGTGTGTTCTTGGGCATC
Il4	NM_021283.1	TGCTTGAAGAAGAACTCTAGTGTTCTCATGGAGCTGCAGAGACTCTTTCG GGCTTTTCGATGCCTGGATTCATCGATAAGCTGCACCATGAATGAGTCCA
IL4r	NM_001008700.3	CCCACAGCAGTGCTGACGTTCCTAAGTCCTGGGCTTTCCTAGCTGATGTT GTCCTACCTACTCAGTCCCATTTTGTCCACCGAATAGACCTGTCACTCAA
Il6	NM_031168.1	CTCTCTGCAAGAGACTTCCATCCAGTTGCCTTCTTGGGACTGATGCTGGT GACAACCACGGCCTTCCCTACTTCACAAGTCCGGAGAGGAGACTTCACAG
Il10	NM_010548.1	GGGCCCTTTGCTATGGTGTCCTTTCAATTGCTCTCATCCCTGAGTTCAGA GCTCCTAAGAGAGTTGTGAAGAAACTCATGGGTCTTGGGAAGAGAAACCA
Il10ra	NM_008348.2	TGTTGTCGCGTTTGCTCCCATTCCTCGTCACGATCTCCAGCCTGAGCCTA GAATTCATTGCATACGGGACAGAACTGCCAAGCCCTTCCTATGTGTGGTT
Il10rb	NM_008349.5	CTTTACACCTGCGTTTCTCAGCCCCACAAATTGAGAATGAGCCTGAGACG TGGACCTTGAAGAACATTTATGACTCATGGGCTTACAGAGTGCAATACTG
Il13ra1	NM_133990.4	CTCAAACCGACCGACATAATATTTTAGAGGTTGAAGAGGACAAATGCCAG AATTCCGAATCTGATAGAAACATGGAGGGTACAAGTTGTTTCCAACTCCC
Itgam (Cd11b)	NM_001082960.1	ATCCCTGTTCAGATCAACAATGTGACCGTATGGGATCATCCCCAGGTCAT CTTCTCCCAGAACCTCTCAAGTGCCTGTCACACTGAGCAGAAATCCCCCC
Itgb2	NM_010578.1	CTGTGATAGGTCTAATGGCTTAATTTGTGGAGGCAATGGCGTGTGCAGGT GTCGTGTTTGTGAATGCTATCCCAATTACACTGGCAGTGCATGTGACTGT
Kcna2 (Kv1.2)	NM_008417.4	GTTAACTGATGTCTGATTGAAGCCTACTAATGTACTCACAGCTCAACAGG ACTGATGCAGATGTTGCATAATAGCCTGCATTGTAGTCAGTGTTCTACAG
Kcna3 (Kv1.3)	NM_008418.2	CTGTTGGTTATGGTGATATGCACCCAGTGACCATAGGAGGCAAGATTGTG GGCTCTCTTTGTGCCATCGCAGGTGTCTTGACCATTGCATTGCCAGTTCC
Kcna5 (Kv1.5)	NM_145983.2	AAAAAGTATCGCATTCCATGACGCAGGAGCCGTTGAAGTGGTGAGCATTC ACTGTAAGATGGATGTATTCATAGCCAGTTTTCTATACCCAGCAGAGGGA
Kcnj2 (Kir2.1)	NM_008425.4	CTTAAGGCGAGAATCGGAGATATGACTGGCTGATTCCGTCTTTGGAATAC TTACTTTGCTACACAGCCTGACGTTGGTCAGAGGTCCGAGACAGTTATAC
Kcnma1 (BK)	NM_001253361.1	CGCATGTGGTGGGCTTTCTTGGCCTCCTCCATGGTGACTTTCTTCGGGGG CCTCTTCATCATCTTGCTCTGGCGGACGCTCAAGTACCTGTGGACCGTTT
Kcnn3 (SK3)	NM_080466.2	ATTGAGATGATGCATGGGGTGTGTTTAGTTGAGGACGTTAGAGGGTATTG GCCACATTTTGACCTTCATTGAAGATGCAGGTCCCTCCTTCACAGTGCCC
Kcnn4	NM_008433.4	AACTGGCATCGGACTCATGGTTCTGCACGCTGAGATGTTGTGGTTCCTGG

(SK4)		GCTGCAAGTGGGTGCTGTACCTGCTCCTGGTTAAGTGTTTGATCACCCTG
Mrc1<br>(CD206)	NM_008625.1	GTTCCGAAATGTTGAAGGGAAGTGGCTTTGGTTGAACGACAATCCTGTCT<br>CCTTTGTCAACTGGAAAACAGGCGATCCCTCTGGTGAACGGAATGATTGT
Msr1<br>(SR-A)	NM_001113326.1	GATTTCGTCAGTCCAGGAACATGGGAATTCACTGGATGCAATCTCCAAGT<br>CCTTGCAGAGTCTGAATATGACACTGCTTGATGTTCAACTCCATACAGAA
Myc	NM_010849.4	CCCTCAACGTGAACTTCACCAACAGGAACTATGACCTCGACTACGACTCC<br>GTACAGCCCTATTTCATCTGCGACGAGGAAGAGAATTTCTATCACCAGCA
Ncf1	NM_001286037.1	ACCATCCGCAACGCACAGAGCATCCACCAGCGTTCTCGGAAGCGCCTTAG<br>CCAGGACACCTATCGCCGCAACAGCGTCCGATTCCTGCAGCAGCGCAGAC
Nfkbia<br>(IκBα)	NM_010907.1	GTCAGAATTCACAGAGGATGAGCTGCCCTATGATGACTGTGTGTTTGGAG<br>GCCAGCGTCTGACATTATAAGTGGAAAGTGGCAAAAAAGAATGTGGACTT
Nme2<br>(NDPK-B)	NM_001077529.2	GCGGCGGCGGTGGCAGGAACCCACGTGGCTTCCTGGGCCGGCTTGGGTCG<br>GCCGGGCCTCATCGACTACACTTCTTGCCTCCACAGGACCATGGCCAACC
Nos2	NM_010927.3	CCCCCCTCCTCCACCCTACCAAGTAGTATTGTACTATTGTGGACTACTAA<br>ATCTCTCTCCTCTCCTCCCTCCCCTCTCTCCCTTTCCTCCCTTCTTCTCC
Nox1	NM_172203.1	CTCCAAACATGACAGTGATGTATGCAGCATTTACCAGTATTGCTGGCCTT<br>ACTGGAGTGATTGCCACTGTAGCTTTGGTTCTCATGGTAACGTCAGCTAT
Nox4	NM_015760.4	TCCCAGAAAGCTTCTCTTCACAACCATTCCTGGTCTGACGGGTGTCTGCA<br>TGGTGGTGGTATTGTTCCTCATGGTTACAGCTTCTACCTACGCAATAAGA
Nr3c1<br>(GR)	NM_008173.3	ACCAGGATTCAGAAACTTACACCTGGATGACCAAATGACCCTTCTACAGT<br>ACTCATGGATGTTTCTCATGGCATTTGCCCTGGGTTGGAGATCATACAGA
Orai1	NM_175423.3	ACACCGGGCACCCACTATGCCTAAGTCCTCACCTTCCCACTGGCCCTTTG<br>AGGCCTTGGCCTTATGCCCTTCTCCATGACCTTGTCCTGGCCCAGTCCGG
Orai3	NM_198424.3	ACATGTCTGCACAACTGTCCTCAAGGCAATCAGCCCTTGCTTATCTGTAT<br>AATCTGCTTTGCAGTTGGCATCCTGGGAGAGATTTTACAGGGCTCCTCAG
P2rx7	NM_001038887.1	CTGGAGGAACTGGAAGTTAACCGTTCCTGCTGAGAAATCGGTGTGTTTCC<br>TTTGGCTGCTCCTAGGTGAGGGTTTGCTGTGGTCTAGCCTGGGAAGTAGG
P2ry2	NM_008773.3	TAGCCATTTTGTGGCTTACAGCTCCGTCATGCTGGGTCTGCTTTTTGCTG<br>TGCCCTTTTCCGTAATCCTGGTCTGTTACGTGCTTATGGCCAGGCGGCTG
P2ry12	NM_027571.3	GATCACCCAGGTTCTCTTCCCATTGCTGTACACCGTCCTGTTCTTTGCTG<br>GGCTCATCACGAACAGCTTGGCAATGAGGATTTTCTTTCAGATCCGCAGT
Phpt1	NM_029293.2	GAACATGGCGGCGGACCTCGGTCAGATTCCTGATGTAGACATTGATTCGG<br>ATGGCGTCTTCAAGTATGTGCTGATTCGAGTCCACTTAGCAGAGCCTTCT
Pparg	NM_011146.1	ACCAAGTGACTCTGCTCAAGTATGGTGTCCATGAGATCATCTACACGATG<br>CTGGCCTCCCTGATGAATAAAGATGGAGTCCTCATCTCAGAGGGCCAAGG
Ptgs2<br>(Cox2)	NM_011198.3	CCATCAGTTTTTCAAGACAGATCATAAGCGAGGACCTGGGTTCACCCGAG<br>GACTGGGCCATGGAGTGGACTTAAATCACATTTATGGTGAAACTCTGGAC
Ptk2b<br>(Pyk2)	NM_001162365.1	CTTCCGCCGCTTCACAACCGCCAGTGATGTCTGGATGTTTGCTGTATGCA<br>TGTGGGAGATCCTCAGCTTTGGGAAGCAGCCTTTCTTCTGGCTCGAAAAT
Ptpn6<br>(SHP-1)	NM_013545.2	GACCGAGGCCCAGTACAAGTTTATTTACGTGGCCATTGCCCAGTTCATCG<br>AAACGACCAAGAAGAAACTGGAGATCATACAATCCCAGAAGGGCCAGGAG
Rest	NM_011263.1	GAACGCCCGTATAAATGTGAACTTTGTCCTTACTCAAGCTCTCAGAAGAC<br>TCATCTAACGCGACACATGCGGACTCATTCAGGTGAGAAGCCATTTAAAT
Retnla<br>(Fizz1)	NM_020509.3	GAATACTGATGAGACCATAGAGATTATCGTGGAGAATAAGGTCAAGGAAC<br>TTCTTGCCAATCCAGCTAACTATCCCTCCACTGTAACGAAGACTCTCTCT
Socs1	NM_009896.2	CAGCTTGTGTCTGGGGCCAGGACCTGAATTCCACTCCTACCTCTCCATGT<br>TTACATATTCCCAGTATCTTTGCACAAACCAGGGGTCGGGGAGGGTCTCT

Socs3	NM_007707.2	CCGCGACAGCTCGGACCAGCGCCACTTCTTCACGTTGAGCGTCAAGACCC<br>AGTCGGGGACCAAGAACCTACGCATCCAGTGTGAGGGGGGCAGCTTTTCG
Stim1	NM_009287.4	GCTGCTGTCACATCTTCTCCACTTCAGTGCATGTCTTAGTTGCTCTTCCC<br>TCAGTTCCCACTCCACTTTTGGGGTCCAGCTTCTGTCTCTGCTGTCCCAG
Tgfb1	NM_011577.1	GGAGTTGTACGGCAGTGGCTGAACCAAGGAGACGGAATACAGGGCTTTCG<br>ATTCAGCGCTCACTGCTCTTGTGACAGCAAAGATAACAAACTCCACGTGG
Tgfbr1	NM_009370.2	TCAGAAGTAGTGGCCAGCTGTGTCTCTAGTAGGACAGTAAAGGCATGAAG<br>CTCAGCCTGTAATCCTGCTACTACAGTAGTACTCCAGAAGTGCCTTGAGG
Tgfbr2	NM_009371.2	TGTGCAAGTTTTGCGATGTGAGACTGTCCACTTGCGACAACCAGAAGTCC<br>TGCATGAGCAACTGCAGCATCACGGCCATCTGTGAGAAGCCGCATGAAGT
Tlr2	NM_011905.2	GCAGGCGGTCACTGGCAGGAGATGTGTCCGCAATCATAGTTTCTGATGGT<br>GAAGGTTGGACGGCAGTCTCTGCGACCTAGAAGTGGAAAAGATGTCGTTC
Tlr4	NM_021297.2	AACGGCAACTTGGACCTGAGGAGAACAAAACTCTGGGGCCTAAACCCAGT<br>CTGTTTGCAATTAATAAATGCTACAGCTCACCTGGGGCTCTGCTATGGAC
Tnf	NM_013693.1	TTCCTGAGTTCTGCAAAGGGAGAGTGGTCAGGTTGCCTCTGTCTCAGAAT<br>GAGGCTGGATAAGATCTCAGGCCTTCCTACCTTCAGACCTTTCCAGACTC
Tnfrsf1a<br>(TNFR1)	NM_011609.2	CTCCTTGCCAAGCTGACAAGGACACGGTGTGTGGCTGTAAGGAGAACCAG<br>TTCCAACGCTACCTGAGTGAGACACACTTCCAGTGCGTGGACTGCAGCCC
Tnfrsf1b<br>(TNFR2)	NM_011610.3	GTGTGTGTCCATGTTTGCATGTATGTGTGTGCCAGTGTGTGGAGGCCAGA<br>GGTTGGCTTTGGGTGTGTTTGATCACTCTCAGTTACTGAGGCAGGGCTCT
Trem1	NM_021406.5	GTGGCTCCTGTGCTTTCTCTAGGACACACTGGCATTTGCTTGTTTCCACC<br>TCTTTGGGTGGGAAAAGGATGGTAGATCCATTCCTTCTACTTCAGCAGTG
Trem2	NM_031254.2	GGGCGCCTACCCTAGTCCTGACTGTTGCTCAATCCAGGAGCACAGTTCCT<br>GTGGGCTGAGCCTGACTGGCTTGGTCATCTCTTTTCTGCACTTCAAGGGA
Trpm2	NM_138301.2	TGCAGGGAGTGAGCCTCCGGTCCCTCTATAAGCGATCAACAGGCCACGTT<br>ACCTTCACCATTGACCCAGTCCGTGACCTTCTCATTTGGGCCGTTATCCA
Trpm4	NM_175130.4	AGGAGTTGCTGACGGTCTATTCATCAGAAGACGGCTCCGAGGAGTTTGAG<br>ACTATCGTTTTGAGGGCTCTTGTGAAAGCCTGTGGGAGCTCTGAGGCCTC
Trpm7	NM_021450.2	CAAATTCAATTCTGGGAGTGAAGAGAGAATCCGGGTCACTTTTGAAAGAG<br>TGGAGCAGATGAGCATTCAGATTAAAGAAGTTGGAGATCGTGTCAACTAC
Tspo	NM_009775.4	GACACTGGCTCCCATCTGGGGCACACTGTATTCAGCCATGGGGTATGGCT<br>CCTACATAGTCTGGAAAGAGCTGGGAGGTTTCACAGAGGACGCTATGGTT
