# Supplementary material for: Comparing Effects of Transforming Growth Factor β1 on Microglia From Rat and Mouse: Transcriptional Profiles and Potassium Channels
Source: Front Cell Neurosci. 2018 May 3;12:115. doi: 10.3389/fncel.2018.00115 (PMC5946019; doi:10.3389/fncel.2018.00115)
Supplement: Supplementary file 3 [file Table_3.PDF]

Comparing effects of transforming growth factor b1 on microglia from rat and mouse: Transcriptional profiles and potassium channels

Starlee Lively, Doris Lam, Raymond Wong and Lyanne C. Schlichter*
* Correspondence: Professor Lyanne C. Schlichter Lyanne.Schlichter@uhnresearch.ca

Supplementary Table 3. Genes with unchanged expression after TGFβ1 treatment
Rat and mouse microglia were stimulated for 24 h with TGFβ1. To show differences in basal expression between species, unstimulated (control) mRNA levels are expressed as mean counts ± SEM (*n*=4–6 individual cultures). Effects of TGFβ1 on a given gene are expressed as fold changes with respect to species-matched control levels. Bold numbers and asterisks indicate species differences in control mRNA counts.

		Control			TGFβ1	
		mRNA counts ± SD			*Fold change with respect to Control*	
Category	Gene	Rat	Mouse		Rat	Mouse
Microglia markers and immune modulators	*Aif*	**26159 ± 4131** ***	1 ± 1		1.03	6.63
	Cd68	**48597 ± 2923** ***	9206 ± 596		0.93	1.04
	Nfkbia	**5590 ± 2472** ***	1167 ± 264		0.54	0.64
	Socs3	60 ± 34	9 ± 5		0.58	1.43
	Tlr2	**3340 ± 1329** ***	360 ± 115		0.81	1.61
	Tspo	**2212 ± 1054** **	722 ± 154		1.45	1.41
Anti-inflammatory mediators	*Arg1*	7 ± 4	22 ± 21		0.83	0.96
	Ccl22	9 ± 6	10 ± 5		0.97	0.47
	Cd163	7 ± 4	3 ± 3		0.48	3.16
	Il10ra	**895 ± 99** ***	451 ± 87		0.89	1.00
	Il10rb	**1595 ± 151** ***	2438 ± 251		0.98	0.91
	Retnla	6 ± 4	16 ± 7		1.26	1.07
Pro-inflammatory mediators	*Ccr2*	10 ± 6	31 ± 12		0.67	0.73
	Ifng	6 ± 4	3 ± 2		0.80	2.20
	Ifngr2	29 ± 11	**1817 ± 192** ***		1.37	1.10
	Il1r1	11 ± 5	12 ± 6		0.89	2.07
	Il6	10 ± 6	10 ± 2		2.92	1.64
	Nos2	43 ± 32	26 ± 21		0.32	1.50
	Ptgs2	21 ± 16	60 ± 30		0.73	0.71
	Ptk2b	1333 ± 296	1028 ± 144		0.89	1.08
	Tnfrsf1a	**1064 ± 46** **	922 ± 44		0.94	0.99
Microglial physiology	*Adora1*	5 ± 5	10 ± 2		1.36	0.39
	Adora2a	41 ± 40	17 ± 6		0.51	1.09
	Cybb	3426 ± 605	**8402 ± 2132** ***		0.91	0.80
	Hvcn1	**2240 ± 266** ***	268 ± 83		1.14	1.64
	Fcgr1	**6196 ± 2179** ***	1436 ± 203		0.74	0.70

	Msr1	**6768 ± 1434** **	3528 ± 564		0.82	0.87
	Nox4	2 ± 1	6 ± 3		1.74	1.93
	P2ry2	69 ± 13	62 ± 17		1.33	1.57
Ion channels and their regulators	*Calm*	**21304 ± 3538** ***	579 ± 40		0.90	0.88
	Kcna2	**73 ± 47** *	10 ± 6		0.64	1.04
	Kcnj2	**1913 ± 657** ***	422 ± 90		0.66	0.78
	Kcnma1	6 ± 3	30 ± 10		2.45	1.34
	Nme2	**4933 ± 482** ***	104 ± 20		1.08	1.27
	Orai1	**1036 ± 177** ***	138 ± 12		0.97	1.29
	Phtp1	**946 ± 63** ***	395 ± 33		1.12	1.05
	Stim1	**404 ± 37** ***	55 ± 22		1.05	1.59
	Trpm2	**1366 ± 888** ***	37 ± 6		0.81	1.23
	Trpm4	39 ± 5	17 ± 4		1.20	1.33
