# Supplementary material for: Comparing Effects of Transforming Growth Factor β1 on Microglia From Rat and Mouse: Transcriptional Profiles and Potassium Channels
Source: Front Cell Neurosci. 2018 May 3;12:115. doi: 10.3389/fncel.2018.00115 (PMC5946019; doi:10.3389/fncel.2018.00115)
Supplement: Supplementary file 4 [file Table_4.PDF]

Comparing effects of transforming growth factor β1 on microglia from rat and mouse: Transcriptional profiles and potassium channels

Starlee Lively, Doris Lam, Raymond Wong and Lyanne C. Schlichter*
* Correspondence: Professor Lyanne C. Schlichter Lyanne.Schlichter@uhnresearch.ca

Supplementary Table 4. TGFβ1 and IL-10 differentially alter microglial gene expression. Effects of TGFβ1 or IL-10 on a given gene 24 h after stimulation are expressed as fold changes with respect to species-matched control levels. Most data for IL-10 are from Lam et al., 2017, with a few additional genes: *Ccl3*, *Ccr2*, *Ccr5*, *Csf1r*, *Hvcn1*, *Itgb2*, *Kcnma1*, *Kcnn3*, *Kcnn4*, *Nme2*, *Orai1*, *Orai3*, and *Phtp1*. Arrows indicate statistically significant increases (↑) or decreases (↓) in response to TGFβ1 or IL-10 treatment. Bold numbers and asterisks indicate differences in responses to TGFβ1 versus IL-10. One symbol (arrow or asterisk) indicates $p<0.05$; two, $p<0.01$; three, $p<0.001$

		Rat			Mouse	
		Fold change wrt control			*Fold change wrt control*	
Category	Gene	TGFβ1	IL-10		TGFβ1	IL-10
Microglia markers and immune modulators	*Aif1*	1.03	1.06		6.63	1.35
	Cd68	0.93	**1.26** ↑ **		1.04	0.95
	Csf1r	1.29 ↑↑	1.29		1.01	1.17
	Cx3cr1	**13.23** ↑↑ **	0.65 ↓↓		**2.07** ↑↑ **	0.60 ↓↓
	Itgam	**2.25** ↑↑ **	1.54 ↑↑		1.17	**1.82** ↑↑↑ *
	Nfkbia	0.54	0.95		0.64	1.08
	Nr3c1	1.06	1.04		0.76 ↓↓	**1.42** ↑ **
	Socs1	0.39 ↓	0.73		1.10	1.64
	Socs3	0.58	9.44		1.43	**63.51** ↑↑↑ **
	Tlr2	0.81	1.37		1.61	0.84
	Tlr4	1.09	1.49 ↑		0.59 ↓↓	**1.58** ↑ **
	Tspo	1.45	**3.43** ↑↑↑ *		1.41	1.99
Anti-inflammatory mediators	*Arg1*	0.83	0.93		0.96	**6.18** **
	Ccl22	0.97	0.81		0.47	0.64
	Cd163	0.48	0.57		3.16	2.87
	Il1rn	**0.45** ↓ **	3.48		0.50	0.98
	Il4	1.63	1.04		1.97 ↑↑	0.99
	Il4r	1.02	1.55		2.28 ↑↑	**12.24** ↑↑↑ **
	Il10	0.18	1.09		2.28 ↑↑	1.39
	Il10ra	0.89	1.09		1.00	1.09
	Il10rb	0.98	**1.41** ↑↑↑ **		0.91	1.10
	Il13ra1	0.84	1.33		1.38 ↑	2.03 ↑↑↑
	Mrc1	**0.07** ↓↓ **	1.44		0.11 ↓↓	**3.00** ↑↑ **
	Myc	**2.01** ↑↑ **	1.10		0.44 ↓↓	0.61
	Pparg	**0.04** ↓↓ **	0.89		0.35 ↓↓	0.85
	Retnla	1.26	1.11		1.07	**4.70** **

	Tgfb1	**1.41** ↑ *	1.14		0.72 ↓	0.91
	Tgfbr1	**8.07** ↑↑ **	1.14		**2.57** ↑↑ *	0.72
	Tgfbr2	**1.99** ↑↑ **	1.35 ↑↑		1.37	1.57 ↑↑↑
	Trem2	1.40 ↑↑	1.25 ↑		1.03	0.79
Pro-inflammatory mediators	*Casp1*	0.89	1.14		**0.49** ↓↓ **	1.06
	Ccl3	**0.26** ↓↓ **	1.03		0.30 ↓	0.66
	Ccr2	0.67	0.88		0.73	0.73
	Ccr5	**0.37** ↓↓ *	1.52		**0.24** ↓↓ **	5.17
	Ifng	0.80	0.69		2.20	1.61
	Ifngr1	**2.12** ↑↑ **	1.11		**0.50** ↓↓ **	1.07
	Ifngr2	1.37	1.11		1.10	1.11
	Il1b	0.10 ↓↓	1.31		0.54	**4.86** ↑↑↑ **
	Il1r1	0.89	0.88		2.07	1.14
	Il6	**2.92** **	0.79		1.64	0.55
	Nos2	0.32	1.70		1.50	1.25
	Ptgs2	0.73	1.34		0.71	1.08
	Ptk2b	0.89	**1.48** *		1.08	1.05
	Tnfa	**0.25** ↓↓ **	1.08		0.63	0.50
	Tnfrsf1a	0.94	**1.44** **		0.99	**1.59** **
	Tnfrsf1b	**0.39** ↓↓ **	1.28		**1.57** *	0.98
	Trem1	3.45 ↑	3.13		4.05 ↑↑	1.94
Microglial physiology	*Adora1*	1.36	1.80		0.39	0.58
	Adora2a	0.51	1.48		1.09	0.90
	Cybb	0.91	**1.45** **		0.80	0.69
	Fcgr1	0.74	**1.48** *		0.70	**2.38** **
	Fcgr2b	1.45 ↑	**3.57** ↑↑↑ *		2.04 ↑↑	3.93 ↑↑↑
	Fcgr3a	0.18 ↓↓	**2.12** ↑↑↑ **		0.68	**1.92** ↑ *
	Itgb2	1.27 ↑	1.42		1.22	0.88
	Msr1	0.82	1.27		0.87	**1.93** ↑↑↑ **
	Ncf1	**2.76** ↑↑ **	0.94		**1.89** ↑↑ *	1.37
	Nox1	**3.52** ↑↑ **	0.95		2.06 ↑	0.63
	Nox4	1.74	1.76		1.93	0.43
	P2rx7	**2.56** ↑↑ **	1.51		0.97	1.15
	P2ry2	1.33	**2.57** ↑↑ *		1.57	1.30
	P2ry12	1.55 ↑↑	0.95		1.04	1.53 ↑
Ion channels and their regulators	*Calm*	0.90	**1.22** **		0.88	0.98
	Hvcn1	1.14	1.20		**1.64** **	0.82
	Kcna2	0.64	0.89		1.04	0.80
	Kcna3	**7.42** ↑↑ **	1.15		**5.58** ↑↑ **	0.87
	Kcna5	**4.24** ↑↑ *	1.00		3.44 ↑	1.16
	Kcnj2	**0.66** *	1.06		0.78	0.49 ↓

	Kcnma1	**2.45** *	1.10		1.34	0.89
	Kcnn3	0.83	0.65		**2.11** ↑ **	0.82
	Kcnn4	**2.46** ↑↑ **	1.32		0.89	0.62
	Nme2	1.08	1.31		1.27	1.06
	Orai1	0.97	**1.47** *		1.29	1.10
	Orai3	1.35 ↑↑	1.30		1.19	1.20
	Phtp1	1.12	1.08		1.05	0.99
	Ptpn6	0.99	1.15		0.68 ↓	1.07
	Rest	1.25 ↑↑	1.30		1.16 ↑	1.28
	Stim1	1.05	1.15		1.59	1.50
	Trpm2	0.81	0.86		1.23	0.71
	Trpm4	1.20	1.27		1.33	1.91
	Trpm7	1.17 ↑↑	1.15		0.91	**1.15** *
