# Supplementary material for: Comparing Effects of Transforming Growth Factor β1 on Microglia From Rat and Mouse: Transcriptional Profiles and Potassium Channels
Source: Front Cell Neurosci. 2018 May 3;12:115. doi: 10.3389/fncel.2018.00115 (PMC5946019; doi:10.3389/fncel.2018.00115)
Supplement: Supplementary file 5 [file Image_1.PDF]

Comparing effects of transforming growth factor b1 on microglia from rat and mouse: Transcriptional profiles and potassium channels

Starlee Lively, Doris Lam, Raymond Wong and Lyanne C. Schlichter*

*** Correspondence:** Professor Lyanne C. Schlichter
Lyanne.Schlichter@uhnresearch.ca

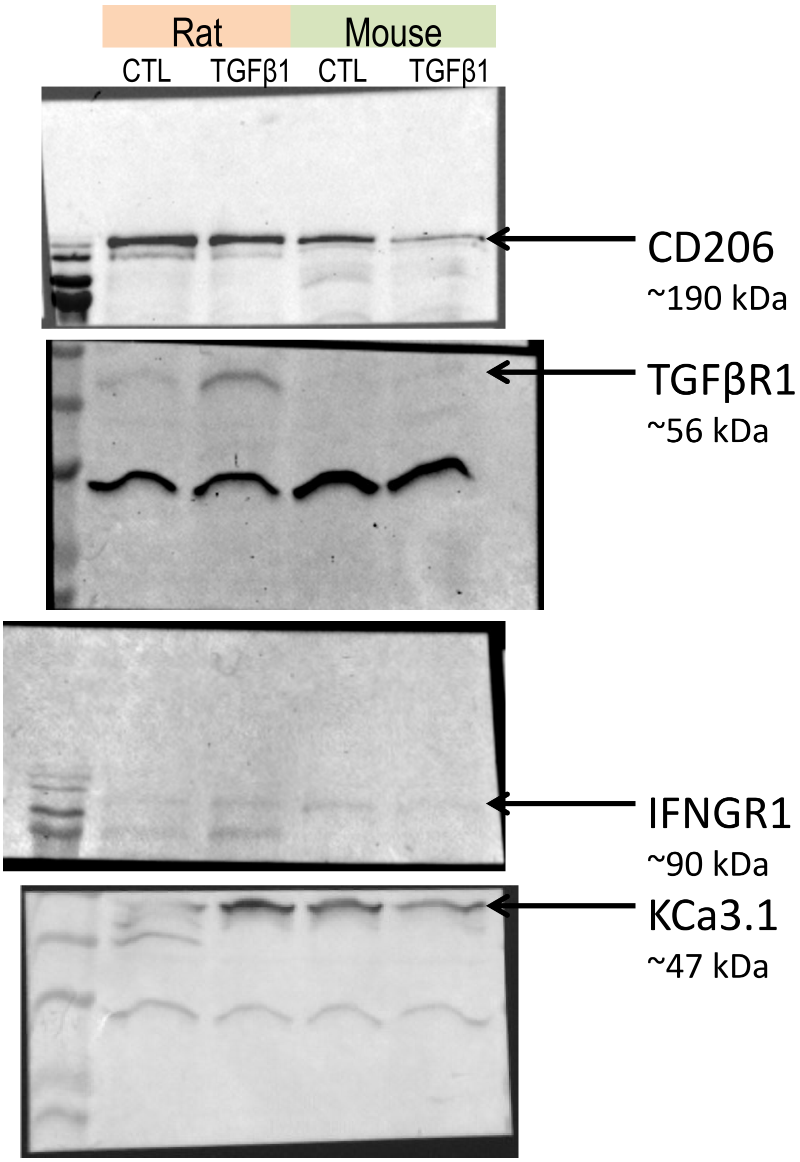

Supplementary Figure 1. Examples of uncropped Western Blots used for quantification. In a pilot study, uncut blots were used to test antibody specificity; however, because samples were limited, blots were cut into strips and sequentially reprobed with a different antibody. For TGFβR1 and KCa3.1, lower band present in all lanes is from a prior exposure with a different antibody not used in the study.
